# L‐Tryptophan Produced by *Bifidobacterium pseudocatenulatum* NCU‐08 Delays Aging in SAMP8 Mice by Activating the Sirt1/P53/P21/Rb Signaling Pathway

**DOI:** 10.1111/acel.70166

**Published:** 2025-07-11

**Authors:** Tangchang Xu, Xiaoyun Wu, Yifei Zhang, Yujie Cai, Xinfeng Zhang, Qingwei Zeng, Jie Luo, Jing Wei, Tingtao Chen

**Affiliations:** ^1^ School of Life Sciences Nanchang University Nanchang China; ^2^ Second College of Clinical Medicine Jiangxi Medical College, Nanchang University Nanchang China; ^3^ Jiangxi Province Key Laboratory of Bioengineering Drugs School of Pharmacy, Jiangxi Medical College, Nanchang University Nanchang China; ^4^ School of Public Health Jiangxi Medical College, Nanchang University Nanchang China; ^5^ China National Engineering Research Center for Bioengineering Drugs and the Technologies Institute of Translational Medicine, Jiangxi Medical College, Nanchang University Nanchang China

**Keywords:** aging, *Bifidobacterium pseudocatenulatum* NCU‐08, gut microbiota, L‐tryptophan, seven centenarians, Sirt1/P53/P21/Rb signaling pathway

## Abstract

Gut microbiota delays aging by regulating the immune, metabolic, and neurological functions of the host. However, current research on novel probiotics with antiaging properties significantly lags, impacting their application in clinical treatments. In this study, metagenomics, culturomics, and probiotic property screening were used to identify *Bifidobacterium pseudocatenulatum* NCU‐08 as a potential probiotic with anti‐aging properties. In addition, *B. pseudocatenulatum* NCU‐08 effectively improved the behavioral characteristics, significantly reduced the levels of the age‐related protein β‐galactosidase (β‐gal) (BP: M = 0.81 vs. 1.13, *p* < 0.05), attenuated neuronal damage in the hippocampus, and improved the composition of the gut microbiota of senescence‐accelerated mouse tendency‐8 (SAMP8) mice. The targeted metabolomics suggested that L‐tryptophan (L‐Trp) may be a key substance for *B. pseudocatenulatum* NCU‐08 to exert anti‐aging effects (BP: M = 14878.6 ng/mL vs. 5464.99 ng/mL, *p* < 0.01). Mechanistically, using the aging model of SAMP8 mice and HT22 mouse hippocampal neuronal cells, it was found that *B. pseudocatenulatum* NCU‐08 might enter the intestine to regulate L‐Trp, and then transport it to the brain. In the brain, L‐Trp was metabolized to NAD^+^, which activated the Sirt1/P53/P21/Rb signaling pathway, thereby exerting antiaging effects. Interestingly, this antiaging effect was inhibited after the intervention of the Sirt1 inhibitor EX‐527. This study is the first to confirm the antiaging properties of NCU‐08 isolated from the fecal samples of seven centenarians in Jiangxi Province, providing data support for the future development of probiotic preparations with antiaging effects.

## Graphical abstract

Bifidobacterium pseudocatenulatum NCU‐08, isolated from the feces of centenarians, facilitated the entry of L‐tryptophan into hippocampal neurons of SAMP8 mice, where it was metabolized into NAD+. The increased NAD+ levels activated Sirt1 protein, thereby suppressing the expression of senescence‐associated proteins P53, P21, and Rb, ultimately delaying aging.

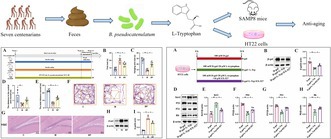

## 1 Introduction

Aging is an irreversible process primarily characterized by a decline in the functions of tissues and organs, which severely impacts the health of older adults (Guo et al. 2022). Currently, global population aging is intensifying, with an anticipated population of over 1.4 billion individuals aged 60 and above by 2030 (Stewart and Sharples 2022). As the incidence and mortality rates of age‐related diseases such as cancer, cardiovascular disease, diabetes, and neurodegenerative disorders continue to rise, many countries and regions are facing significant economic pressures and social burdens (Ni et al. 2023). Despite the promising results shown by antiaging drugs like metformin, rapamycin, and resveratrol in various animal models, their adverse effects, unclear antiaging mechanisms, and lack of effective clinical trial data hinder their clinical application (Sorrenti et al. 2022; Zhao et al. 2017). Therefore, actively pursuing safer and more effective antiaging strategies is crucial for enhancing the lifespan and quality of life of older adults.

The mechanisms of aging are highly complex, involving multiple factors such as oxidative stress, genetic mutations, and inflammatory responses (Wang et al. 2021, 2024). Recent evidence suggests that the gut microbiota plays an active role in the aging process and is a key factor in healthy aging (DeJong et al. 2020). Studies have found significant differences in the gut microbial composition between centenarians and young adults, as evidenced by increased abundance of *Escherichia coli* , *Clostridium perfringens* , and *Clostridium difficile* , and decreased abundance of *Bacteroides*, *Lactobacillus*, and *Bifidobacterium* (Pang et al. 2023). Additionally, it has been reported that transplanting the fecal microbiota from young mice to old mice can reverse age‐related cognitive dysfunction in those older mice (Pan et al. 2023). Conversely, transferring the fecal microbiota from older mice to younger ones exacerbates aging in the young (D'Amato et al. 2020), indicating that fecal microbiota transplantation (FMT) could be a potential effective intervention against aging. Moreover, the tea polyphenols improved behavioral deficits in aging mice by reshaping the gut microbiota and altering the metabolism of core microbial metabolites, thereby maintaining gut homeostasis via the gut–brain axis (Li, Zhang, et al. 2023). Consequently, targeting the gut microbiota may be an important means of improving the lifespan and quality of life for the elderly.

Probiotics are live microorganisms that confer health benefits to the host when administered in adequate amounts. In recent years, a substantial body of evidence has indicated that probiotics may play a significant role in delaying aging by maintaining the balance of the gut microbiota (Choudhary et al. 2023). Research has found that supplementation with a mixed probiotic (probiotic‐4) significantly improved cognitive deficits, neuronal and synaptic damage, glial activation, and microbial composition in the mouse model of senescence accelerated mouse‐prone 8 (SAMP8) (Yang et al. 2019). Additionally, our research group has confirmed that probiotic combinations ( *Lactobacillus fermentum* SX‐0718, *Lactobacillus casei* SX‐1107, *Bifidobacterium longum* SX‐1326, and *Bifidobacterium animalis* SX‐0582) have been demonstrated to be effective in improving the impaired spatial memory, motor dysfunction, and reduced exploratory behavior of aging SAMP8 mice (Fang et al. 2021). Furthermore, *Lactobacillus plantarum* TY‐Y10 reduced oxidative stress levels in D‐galactose (D‐gal)‐induced aging mice by regulating the gut microbiota and activating hepatic antioxidant pathways via the gut–liver axis (Shi et al. 2024). Thus, probiotic interventions may represent a safe and effective antiaging strategy.

Currently, next‐generation probiotics (NGPs) have emerged as a cutting‐edge research focus in global microbiome studies, demonstrating the potential to overcome the broad‐spectrum limitations of traditional probiotics and achieve precise disease intervention. Among them, *Bifidobacterium pseudocatenulatum* , as a next‐generation probiotic strain, has not yet been included in China's “List of Strains Permitted for Use in Food”. However, it holds significant scientific value and strategic importance in both fundamental research and applied development. It is expected to break through the current limitations of probiotics and open new frontiers for precise nutritional intervention. Notably, *B. pseudocatenulatum* has shown remarkable efficacy in modulating gut microbiota and immune responses (Chen et al. 2021; Moratalla et al. 2016). Wang et al., found that *B. pseudocatenulatum* may be particularly advantageous for improving impaired neural and immune functions identified through gut microbiota analysis in 32 longevity families (Wang et al. 2022). However, the relationship between *B. pseudocatenulatum* and longevity or aging amelioration remains unclear. In this study, we utilized metagenomics, culturomics, and screening for probiotic properties to obtain a potential antiaging strain ( *B. pseudocatenulatum* NCU‐08) from the feces of centenarians. Additionally, at the animal level, we demonstrated that NCU‐08 improved the behavioral characteristics of aged SAMP8 mice, reduced the content of β‐galactosidase in the brain, and altered the composition of the gut microbiota. Meanwhile, NCU‐08 had antiaging effects by producing L‐tryptophan (L‐Trp). Furthermore, we discovered that L‐Trp activated the Sirt1/P53/P21/Rb signaling pathway to delay aging in SAMP8 mice. In summary, our findings provide a theoretical basis and in‐depth insights for the development of clinical antiaging microbial preparations.

## 2 Results

### 2.1 The Probiotic Characteristics of *B. pseudocatenulatum* NCU‐08 in the Feces of Centenarians

**FIGURE 1:**
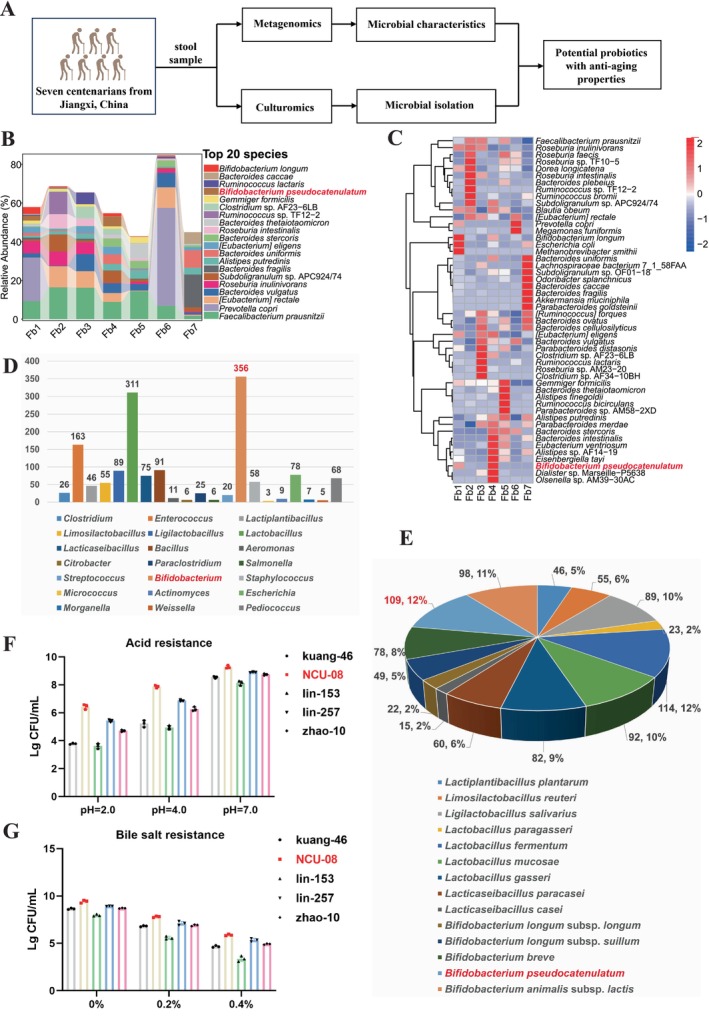
Metagenomics and culturomics were used to identify *B. pseudocatenulatum* NCU‐08 as a potential anti‐aging strain in seven centenarians. (A) Flowchart of experimental design. (B) Taxonomic composition of fecal microbiota at the species level. (C) Heatmap of clustering for differential species at the species level. (D) Isolated microbiota at the genus level. (E) Isolation status of common probiotics. (F) Acid resistance (*n* = 3). (G) Bile salt resistance (*n* = 3). Fb1: Fbkuang; Fb2: Fblai; Fb3: Fblei; Fb4: Fblin; Fb5: Fbliu; Fb6: Fbwang; Fb7: Fbzhao. Data were presented as mean ± SD.

First, seven centenarians (Zhanggong District, Ganzhou City, Jiangxi Province, China) were recruited and performed metagenomic sequencing on their fecal samples (Figure 1A). The participants had an average age of 102.3 years old, an average height of 1.55 m, an average weight of 44.0 kg, and an average BMI of 18.23 (Table S1). Firmicutes and Bacteroidetes were predominant in the gut microbiota of these participants at the phylum level (Figure S1A,B). There were significant variations in the gut microbiota of the seven centenarians, with *Bifidobacterium* being highly enriched in the feces of the Fb1 and Fb4 at the genus level (Figure S1C,D). Notably, *B. pseudocatenulatum* exhibited the most pronounced enrichment at the species level (Figure 1B,C). To verify the prominence of *B. pseudocatenulatum* in the intestines of centenarians, the microorganisms from their feces were isolated. In total, the 21 genera and 38 species, yielding 1508 strains, were isolated (including duplicate strains) (Table S2). Among these, the isolated *Lactobacillus* and *Bifidobacterium* constituted approximately 44% of the total, with *Bifidobacterium* being particularly prominent (Figure 1D). Additionally, the commonly isolated probiotics were analyzed and found that *B. pseudocatenulatum* accounted for 109 strains, or 12%, all sourced from the feces of the seven centenarians (Figure 1E), indicating its high enrichment in the gut of these centenarians.

Subsequently, in order to identify an excellent strain of *B. pseudocatenulatum* for antiaging research in animals, we screened the 109 strains based on their growth performance, acid resistance, and bile salt resistance. The growth performance assessment revealed that the OD_600_ nm for the 109 strains after 48 h of growth ranged from 1.477 to 2.236 (Table S3). The top five strains with growth performance (kuang‐46, NCU‐08, lin‐153, lin‐257, and zhao‐10) were selected for acid resistance and bile salt tolerance, which indicated that NCU‐08 exhibited superior tolerance compared to the other four strains (Figure 1F,G). At the same time, the morphological characteristics of NCU‐08 were observed, which appeared as pale white, raised granules on the agar plates with smooth edges (Figure S2A). Gram staining results revealed that it exhibited bifidobacterial morphology and was gram‐positive (Figure S2B). The sequence alignment with 16S rRNA sequences from the database (Table S4) was predominantly identified as *B. pseudocatenulatum* (Figure S2C). In the Neighbor‐Joining phylogenetic tree, the Bootstrap values revealed that NCU‐08 strain clustered with *B. pseudocatenulatum* B1279 on the same branch, with a sequence similarity of 100% and a confidence level of 52% after 1000 repetitions (Figure S2D). Based on morphological and phylogenetic analysis results, the NCU‐08 strain was identified as *B. pseudocatenulatum* . Additionally, probiotic properties showed that NCU‐08 exhibited good acid production, salt tolerance, and high‐temperature resistance (Figure S2E−G).

### 2.2 The Probiotic *B. pseudocatenulatum* NCU‐08 Effectively Ameliorated Aging in SAMP8 Mice

**FIGURE 2:**
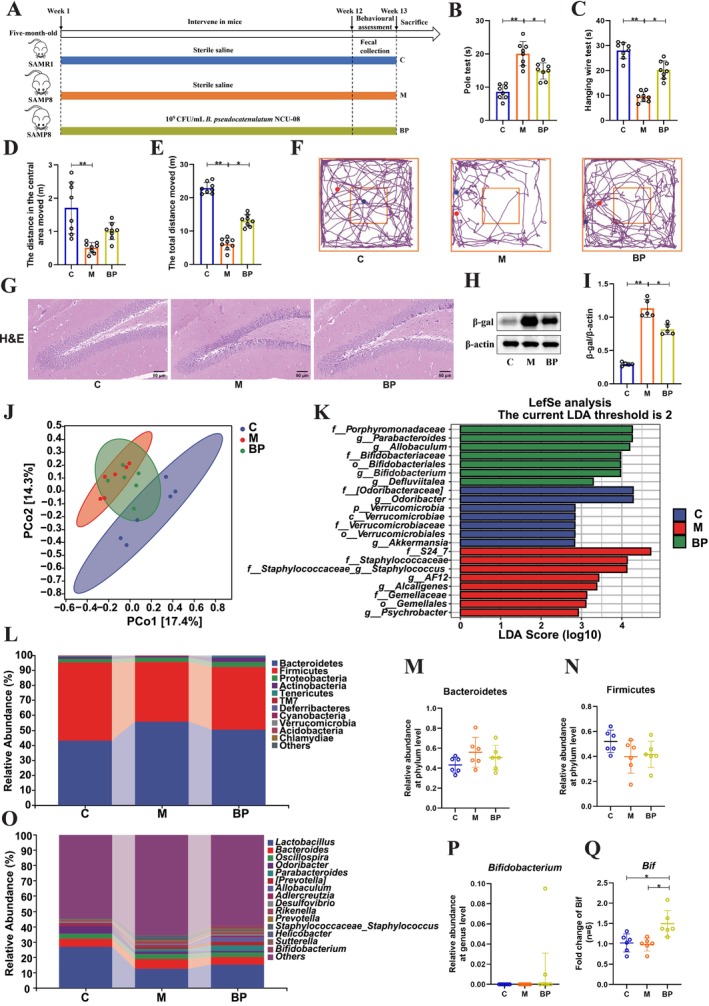
*B. pseudocatenulatum* NCU‐08 significantly delayed aging in SAMP8 mice. (A) Flowchart of experimental animals. (B) Pole test (s). (C) Hanging wire test (s). (D) The distance to move into the central area in 5 min (m). (E) The total distance to move in 5 min (m). (F) The trajectories of movement by mice. (G) H&E staining of the hippocampus region (50 μm). (H) Western blot analysis of the protein of β‐gal. (I) Quantification of β‐gal. (J) Principal Coordinate Analysis (PCoA). (K) Linear Discriminant Analysis (LEFSe) analysis. (L) Taxonomic composition at the phylum level. (M) Relative abundance of Bacteroidetes. (N) Relative abundance of Firmicutes. (O) Microbial taxonomic composition at the genus level. (P) Relative abundance of *Bifidobacterium*. (Q) RT‐qPCR detection of *Bif* (*Bifidobacterium*) expression in feces. β‐gal: β‐galactosidase. C: Control group. M: Model group. BP: *B. pseudocatenulatum* NCU‐08 group. Data were presented as mean ± SD or median (interquartile range), and analyzed using one‐way repeated measures ANOVA, followed by Tukey's test for multiple comparisons. Significance levels are indicated as *p < 0.05 and **p < 0.01.

To evaluate the antiaging effects of *B. pseudocatenulatum* NCU‐08, a 12‐week intervention study utilizing SAMP8 mice was conducted (Figure 2A). Results from the pole test indicated that *B. pseudocatenulatum* NCU‐08 significantly improved the motor abilities of SAMP8 mice (BP: M = 14.84 s vs. 20.05 s, *p* < 0.05) (Figure 2B), enhanced muscle strength and balance (BP: M = 20.27 s vs. 9.44 s, *p* < 0.05) (Figure 2C), increased both the distance traveled to enter the central zone (BP: M = 1.01 m vs. 0.51 m), and total traveled distance (BP: M = 13.14 m vs. 6.14 m, *p* < 0.05), and boosted the exploratory abilities of the mice (Figure 2D−F). These findings suggest that *B. pseudocatenulatum* NCU‐08 can effectively improve the motor dysfunction in SAMP8 mice. Meanwhile, H&E staining results revealed severe neuronal damage in the hippocampal region from the mice of the M group, which was mitigated by *B. pseudocatenulatum* NCU‐08 treatment (Figure 2G). Previous studies have indicated that β‐galactosidase (β‐gal) is a commonly used aging biomarker to assess the degree of aging in animals and cells (Rojas‐Vázquez et al. 2024). Western blot results demonstrated that *B. pseudocatenulatum* NCU‐08 significantly reduced the expression of β‐gal (BP: M = 0.81 vs. 1.13, *p* < 0.05) (Figure 2H−I). Collectively, these results indicate that *B. pseudocatenulatum* NCU‐08 effectively improves aging in SAMP8 mice.

In recent years, gut microbiota has been identified as a key factor in the antiaging process, with imbalances potentially leading to various age‐related degenerative diseases (Du et al. 2021). The high‐throughput sequencing was utilized to analyze the gut microbial composition in mice feces following *B. pseudocatenulatum* NCU‐08 intervention. Results showed that compared to the C group, the α‐diversity (Chao1 index and Simpson index) in the M group was increased. However, *B. pseudocatenulatum* NCU‐08 treatment resulted in a decrease in Simpson index (Figure S3A). Venn diagram analysis revealed that all groups shared 377 common operational taxonomic units (OTUs), while the unique OTUs were 273 for the C group, 301 for the M group, and 325 for the BP group (Figure S3B). Principal coordinates analysis (PCoA) showed no overlap between the sample points of the M group and C group, whereas the BP group samples intersected with both, indicating that *B. pseudocatenulatum* NCU‐08 altered the β‐diversity to some extent (Figure 2J). Furthermore, LEFSe analysis emphasized that distinct differential species existed only among the C group, M group, and BP group (Figure 2K). At the phylum level, Bacteroidetes and Firmicutes dominated the microbiota, with a relative abundance decrease of Bacteroidetes in the BP group compared to the M group, while Firmicutes showed the opposite trend (Figure 2L−N). Similarly, *Lactobacillus* was notably predominant at the genus level. In comparison to the M group, the relative abundance of *Lactobacillus* and *Bifidobacterium* was higher in the BP group, although the difference was not statistically significant (Figure 2O−P). Additionally, to further assess *Bifidobacterium* levels, a significant increase was found by RT‐qPCR in the BP group compared to the M group (Figure 2Q). Thus, these results indicate that *B. pseudocatenulatum* NCU‐08 can partially ameliorate the gut microbial composition of aging SAMP8 mice.

**FIGURE 3:**
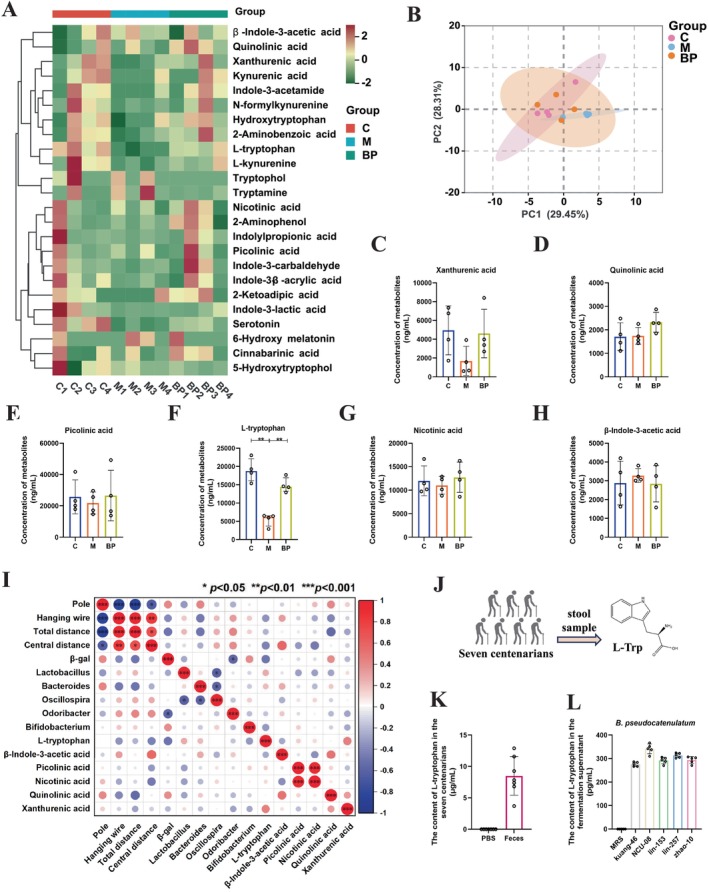
*B. pseudocatenulatum* NCU‐08 may delay aging by L‐tryptophan. (A) Clustered heatmap of tryptophan metabolism in feces. (B) PCA analysis. (C–H) Levels of the top six substances in tryptophan metabolism in feces. (I) Correlation heat map of senescent phenotype and microbiota, metabolites. (J) Schematic diagram of L‐tryptophan detection. (K) Levels of L‐tryptophan in feces of seven centenarians. (L) Levels of L‐tryptophan in the fermentation supernatant of five strains from *B. pseudocatenulatum* after 48 h of growth. MRS: De Man, Rogosa and Sharpe (MRS) broth. C: Control group. M: Model group. BP: *B. pseudocatenulatum* NCU‐08 group. Data were presented as mean ± SD or median (interquartile range), and analyzed using one‐way ANOVA for statistical significance, followed by Tukey's test for multiple comparisons. Significance levels were indicated as *p < 0.05, **p < 0.01, and ***p < 0.001.

Numerous studies have indicated that abnormal tryptophan metabolism can lead to various age‐related diseases during normal aging, including chronic inflammation, atherosclerosis, neurodegeneration, and cancer (Dang et al. 2023). Through metabolomic analysis of tryptophan in mice feces, a total of 31 tryptophan metabolites were detected, with six metabolites not being detected (Table S5). A clustering heatmap revealed that the majority of metabolites in the M group exhibited a decreasing trend compared to the C group, while the BP group showed increased metabolite levels (Figure 3A). PCA analysis indicated no significant difference between the two groups (Figure 3B). Quantitative analysis of the six major metabolites (xanthurenic acid, quinolinic acid, picolinic acid, L‐Trp, nicotinic acid, and β‐indole‐3‐acetic acid) revealed that only the level of tryptophan significantly increased following *B. pseudocatenulatum* NCU‐08 intervention (BP: M = 14878.6 ng/mL vs. 5464.99 ng/mL, *p* < 0.01), suggesting that L‐Trp might be a potential key substance exerting anti‐aging functions (Figure 3C−H). Additionally, correlation heatmap analysis illustrated a negative correlation between L‐Trp and some aging‐related phenotypes (Figure 3I), indicating the involvement of L‐Trp in the antiaging process. Interestingly, the content of L‐Trp in the feces of centenarians was notably high, reaching 8.49 μg/mL (Figure 3J−K), and the content of L‐Trp is most significant in *B. pseudocatenulatum* NCU‐08 (Figure 3L). In addition, in order to explore the antiaging effects of three significantly altered metabolites, including L‐Trp, xanthurenic acid (XA), and indole‐3‐lactic acid (ILA), D‐galactose was used to construct a cellular senescence model. The results showed that compared with the D‐gal group, L‐Trp alone significantly increased the activity of cells (*p* < 0.01) and decreased the expression of *β‐galactosidase* gene (*p* < 0.01) (Figure S3C,D). In summary, L‐Trp may be the critical substance that *B. pseudocatenulatum* NCU‐08 shows antiaging effects.

### 2.3 L‐Trp Improved Aging in SAMP8 Mice

**FIGURE 4:**
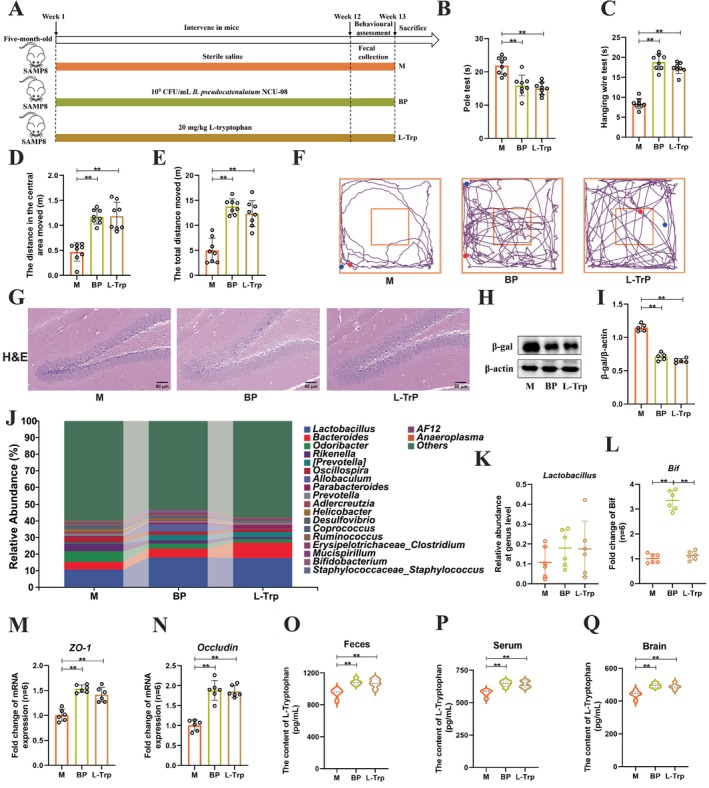
The L‐tryptophan significantly improved the aging characteristics of SAMP8 mice. (A) Flowchart of experimental animals. (B) Pole test (s). (C) Hanging wire test (s). (D) The distance to move into the central area in 5 min (m). (E) The total distance to move in 5 min (m). (F) The trajectories of movement by mice. (G) H&E staining of the hippocampus region (50 μm). (H) Western blot analysis of the protein of β‐gal. (I) Quantification of β‐gal. (J) Microbial taxonomic composition at the genus level. (K) Relative abundance of *Lactobacillus*. (L) RT‐qPCR detection of *Bif* (*Bifidobacterium*) expression in feces. (M) The mRNA level of *ZO‐1* in intestinal tissue. (N) The mRNA level of *Occludin* in intestinal tissue. (O) The content of L‐tryptophan in feces (*n* = 5). (P) The content of L‐tryptophan in serum (*n* = 5). (Q) The content of L‐tryptophan in brain tissue (*n* = 5). M: Model group. BP: *B. pseudocatenulatum* NCU‐08 group. L‐Trp: L‐tryptophan group. Data were presented as mean ± SD, and analyzed using one‐way ANOVA for statistical significance, followed by Tukey's test for multiple comparisons. Significance levels were indicated as **p < 0.01.

To investigate the anti‐aging effects of L‐Trp, the L‐Trp intervention for 12 weeks to mice was administered (Figure 4A). Following the L‐Trp intervention, we examined relevant aging phenotypes similar to those assessed for *B. pseudocatenulatum* NCU‐08, including the behavioral characteristics, the aging‐related protein β‐gal, neuronal damage in the hippocampus, and the composition of gut microbiota. The results showed that L‐Trp significantly improved the motor abilities of SAMP8 mice (L‐Trp: M = 15.06 s vs. 21.89 s, *p* < 0.01) (Figure 4B) and enhanced muscle strength and balance (L‐Trp: M = 17.29 s vs. 8.41 s, *p* < 0.01) (Figure 4C). L‐Trp also increased the distance traveled to enter the central zone (L‐Trp: M = 1.18 m vs. 0.47 m, *p* < 0.01) (Figure 4D) and total traveled distance (L‐Trp: M = 12.36 m vs. 4.99 m, *p* < 0.01) (Figure 4E). Moreover, compared to the M group, L‐Trp exhibited significantly increased movement trajectories (Figure 4F), reduced neuronal damage in the hippocampus (Figure 4G), and a significant decline in the aging protein β‐gal (L‐Trp: M = 0.65 vs. 1.14, *p* < 0.01) (Figure 4H,I). Notably, the therapeutic effects of L‐Trp were comparable to those of *B. pseudocatenulatum* NCU‐08, with no statistical significance between the two. The impact of L‐Trp on gut microbial composition was evaluated. The results indicated that compared to the M and BP groups, L‐Trp did not alter the Simpson index (Figure S4A) or β‐diversity (Figure S4B). Additionally, the Venn diagram revealed that all groups shared 408 common OTUs, while the M, BP, and L‐Trp groups had unique OTUs of 314, 230, and 187, respectively (Figure S4C). LEFSe analysis highlighted that the number of differential species in the BP and L‐Trp groups was lower than that in the M group (Figure S4D). The microbiota was primarily composed of Bacteroidetes and Firmicutes, with a significant decrease in Firmicutes observed at the phylum level (Figure S4E,F). At the genus level, both the BP and L‐Trp groups showed an increase in *Lactobacillus* compared to the C group, and a significant increase in the relative abundance of *Bifidobacterium* was observed in both L‐Trp and *B. pseudocatenulatum* NCU‐08 treatment (Figure 4J–L). These findings suggest that L‐Trp alters the gut microbial composition in SAMP8 mice. Moreover, to explore whether L‐Trp modulates aging, the indicators were assessed related to intestinal barrier function and the distribution of L‐Trp in the gut, serum, and brain tissues. Results indicated that both L‐Trp and *B. pseudocatenulatum* NCU‐08 increased tight junction genes (Figure 4M,N), and L‐Trp levels in the gut, serum, and brain were significantly higher than those in the M group (Figure 4O–Q). In conclusion, these results indicate that L‐Trp improves aging in SAMP8 mice.

### 2.4 L‐Trp Alleviated D‐Galactose‐Induced Aging of HT22 Cells In Vitro

**FIGURE 5:**
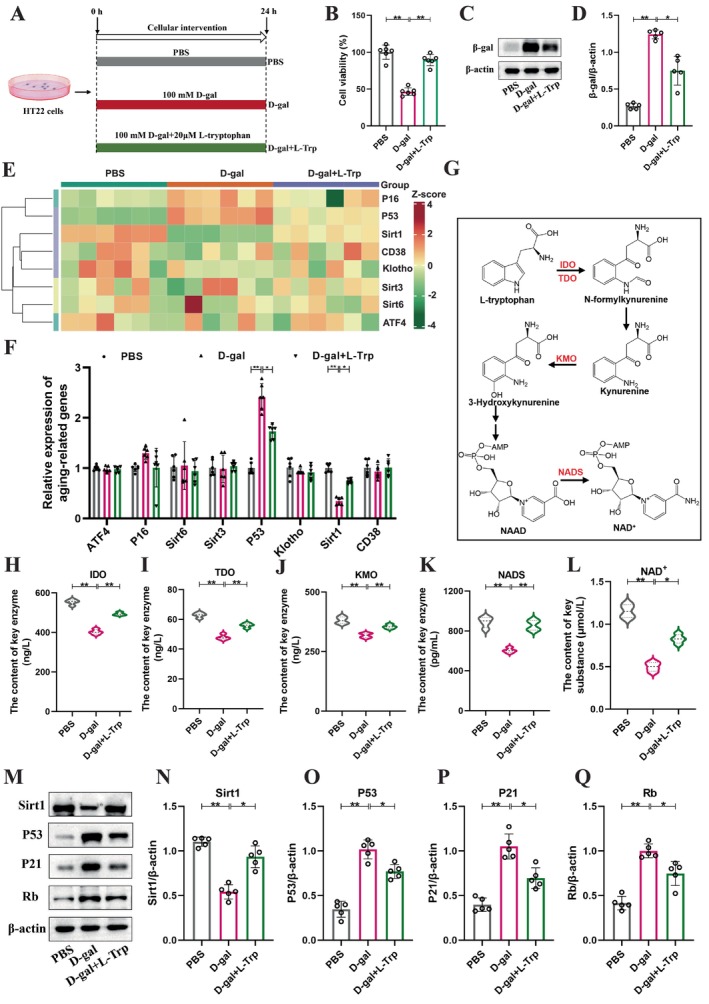
L‐Trp may improve the aging HT22 cell model by regulating the Sirt1/P53/P21/Rb signaling pathway. (A) Flowchart of the cellular experiment. (B) Cell viability test. (C) Western blot analysis of β‐gal. (D) Quantification of β‐gal. (E) Heat map of the relative expression of senescence‐related genes (*ATF4*, *Klotho*, *CD38*, *P16*, *P53*, *Sirt1*, *Sirt3*, and *Sirt6*) in cells by RT‐qPCR. (F) Quantification of relative expression of aging‐related genes (*n* = 6). (G) Schematic diagram of L‐Trp being catabolized by a series of enzymes to produce NAD^+^. (H–K) Levels of key enzymes IDO, TDO, KMO, and NADS in the tryptophan metabolism pathway (*n* = 4). (L) The content of NAD^+^ (*n* = 4). (M) Western blot of key protein in the Sirt1/P53/P21/Rb signaling pathway. (N–Q) Quantifications of key proteins (*n* = 5). L‐Trp: L‐Tryptophan. IDO: Indoleamine 2,3‐dioxygenase. TDO: Tryptophan 2,3‐dioxygenase. KMO: Kynurenine 3‐monooxygenase. NADS: NAD synthetase. NAD^+^: Nicotinamide adenine dinucleotide. PBS: Control group. D‐gal: D‐gal group. D‐gal+ L‐Trp: D‐gal+ L‐tryptophan group. Data were presented as mean ± SD, and analyzed using one‐way ANOVA for statistical significance, followed by Tukey's test for multiple comparisons. Significance levels were indicated as *p < 0.05 and **p < 0.01.

To explore the mechanism of L‐Trp in improving aging, the preliminary explorations at the cellular level were conducted. Based on previous studies (Kwon et al. 2023), D‐gal to induce senescence in HT22 mouse hippocampal neuronal cells was used (Figure 5A). Following L‐Trp treatment, cell viability was significantly improved (D‐gal + L‐Trp: D‐gal = 89.43% vs. 46.44%, *p* < 0.01) (Figure 5B), and the β‐gal protein showed a significant decline (D‐gal + L‐Trp: D‐gal = 0.75 vs. 1.24, *p* < 0.05) (Figure 5C,D). By investigating the existing literature, several common genes closely related to aging were selected, such as the Sirtuin family, *P16*, *P53*, *CD38*, *Klotho*, and *ATF4*, to assess their expression. Clustering heatmap and RT‐qPCR indicated that *P53* gene expression significantly decreased following L‐Trp treatment compared to the D‐gal group (D‐gal + L‐Trp: D‐gal = 1.73 vs. 2.41, *p* < 0.05), while *Sirt1* gene expression significantly increased (D‐gal + L‐Trp: D‐gal = 0.74 vs. 0.34, *p* < 0.05) (Figure 5E,F). Previous research has shown that L‐Trp can be converted to NAD^+^ through the kynurenine pathway (Figure 5G), thereby activating *Sirt1* expression, which in turn modulates the P53/P21/Rb signaling pathway to delay aging (Lei et al. 2024; Szot et al. 2021). Consequently, the key enzymes (IDO, TDO, KMO, and NADS) and NAD^+^ were examined in the tryptophan metabolic pathway, revealing that their levels significantly increased following L‐Trp intervention compared to the D‐gal group (Figure 5H–L). Moreover, the expression of key proteins within the Sirt1/P53/P21/Rb signaling pathway was assessed, finding a significant increase in Sirt1 protein levels following L‐Trp treatment (D‐gal + L‐Trp: D‐gal = 0.93 vs. 0.54, *p* < 0.05), alongside significant decreases in the aging protein of P53 (D‐gal + L‐Trp: D‐gal = 0.77 vs. 1.01, *p* < 0.05), P21 (D‐gal + L‐Trp: D‐gal =0.69 vs. 1.05, *p* < 0.05), and Rb (D‐gal + L‐Trp: D‐gal =0.74 vs. 1.00, *p* < 0.05) (Figure 5M–Q). These results suggest that L‐Trp may exert its antiaging effects by generating NAD^+^ through catabolism to regulate the Sirt1/P53/P21/Rb signaling pathway.

### 2.5 *B. pseudocatenulatum* NCU‐08 and L‐Trp Delayed Aging by Regulating the Sirt1/P53/P21/Rb Signaling Pathway

**FIGURE 6:**
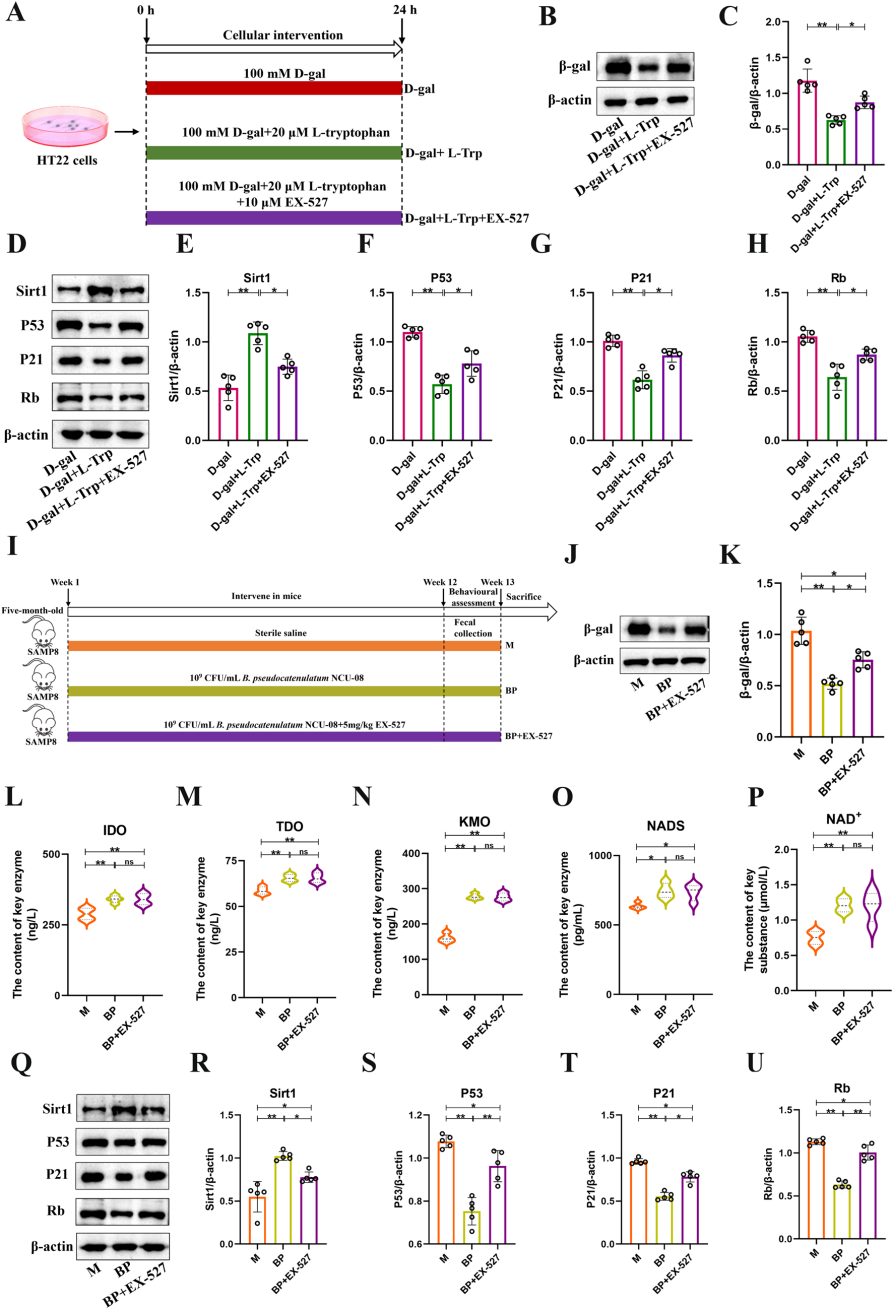
The inhibitor of EX‐527 confirmed that L‐tryptophan can ameliorate cellular senescence by regulating the Sirt1/P53/P21/Rb signaling pathway at the cellular and animal level. (A) Flowchart of the cellular experiment. (B) Western blot analysis of β‐gal. (C) Quantification of β‐gal. (D) Western blot of Sirt1, P53, P21 and Rb protein. (E–H) Quantification of Sirt1, P53, P21 and Rb (*n* = 5). (I) Flowchart of experimental animals. (J) Western blot analysis of the protein of β‐gal. (K) Quantification of β‐gal. (L–O) Levels of key enzymes IDO, TDO, KMO, and NADS in the tryptophan metabolism pathway (*n* = 4). (P) The content of NAD^+^ (*n* = 4). (Q) Western blot of Sirt1, P53, P21 and Rb protein. (R‐U) Quantification of Sirt1, P53, P21 and Rb protein (*n* = 5). IDO: Indoleamine 2,3‐dioxygenase. TDO: Tryptophan 2,3‐dioxygenase. KMO: Kynurenine 3‐monooxygenase. NADS: NAD synthetase. NAD^+^: Nicotinamide adenine dinucleotide. D‐gal: D‐gal group. D‐gal + L‐Trp: D‐gal + L‐tryptophan group. D‐gal + L‐Trp + EX‐527: D‐gal + L‐tryptophan + EX‐527 group. M: Model group. BP: *B. pseudocatenulatum* NCU‐08 group. BP + EX‐527: *B. pseudocatenulatum* NCU‐08 + EX‐527 group. Data were presented as mean ± SD, and analyzed using one‐way ANOVA for statistical significance, followed by Tukey's test for multiple comparisons. Significance levels were indicated as *p < 0.05 and **p < 0.01. “ns” was no significant.

To further validate the role of the Sirt1/P53/P21/Rb signaling pathway in the antiaging effects of L‐Trp, the Sirt1 inhibitor EX‐527 was utilized to intervene in the cells (Figure 6A). The results indicated that compared to the D‐gal + L‐Trp, EX‐527 significantly reduced the expression of β‐gal protein (D‐gal + L‐Trp + EX‐527: D‐gal + L‐Trp = 0.87 vs. 0.62, *p* < 0.05) (Figure 6B,C). Meanwhile, significant changes were found in the expression of key proteins within the Sirt1/P53/P21/Rb signaling pathway following EX‐527 intervention compared to the D‐gal + L‐Trp group, such as a significant decrease in Sirt1 protein (D‐gal + L‐Trp + EX‐527: D‐gal + L‐Trp = 0.74 vs. 1.08, *p* < 0.05), alongside significant increases in P53 (D‐gal + L‐Trp + EX‐527: D‐gal + L‐Trp = 0.77 vs. 0.56, *p* < 0.05), P21 (D‐gal + L‐Trp + EX‐527: D‐gal + L‐Trp =0.86 vs. 0.61, *p* < 0.05), and Rb (D‐gal + L‐Trp + EX‐527: D‐gal + L‐Trp =0.87 vs. 0.64, *p* < 0.05) (Figure 6D–H). Furthermore, as previously assessed, EX‐527 effectively inhibited cell viability (D‐gal + L‐Trp + EX‐527: D‐gal + L‐Trp = 113.20% vs. 191.2%, *p* < 0.01) (Figure S5A), but did not significantly affect the expression of the four enzymes (IDO, TDO, KMO, and NADS) compared to the D‐gal + L‐Trp group, nor did it influence NAD^+^ synthesis (Figure S5B−F). Notably, EX‐527 significantly impacted the transcription levels of Sirt1/P53/P21/Rb signaling pathway‐related factors (Sirt1, P53, P21, and Rb) to varying extents (Figure S5G−J). Thus, the antiaging effect exhibited by L‐Trp at the cellular level through modulation of the Sirt1/P53/P21/Rb signaling pathway is attenuated after the use of EX‐527.

Similarly, EX‐527 was used for validation at the animal level (Figure 6I). Results indicated that, compared to the BP group, EX‐527 significantly increased β‐gal protein expression (BP + EX‐527: BP =0.75 vs. 0.51, *p* < 0.05) (Figure 6J–K). Simultaneously, EX‐527 had no impact on the levels of IDO, TDO, KMO, NADS, and NAD^+^ in the brain tissue (Figure 6L–P). However, following EX‐527 intervention, significant repression of the Sirt1/P53/P21/Rb signaling pathway proteins was observed, with a notable decrease in Sirt1 protein (BP + EX‐527: BP =0.77 vs. 1.02, *p* < 0.05), alongside significant increases in P53 (BP + EX‐527: BP =0.96 vs. 0.75, *p* < 0.01), P21 (BP + EX‐527: BP = 0.78 vs. 0.55, *p* < 0.05), and Rb (BP + EX‐527: BP = 1.00 vs. 0.63, *p* < 0.01) proteins (Figure 6Q–U). Additionally, compared to the BP group, EX‐527 intervention resulted in varying degrees of reduced motor function in the mice. However, hippocampal neuronal damage was unchanged (Figure S6A−F). Concurrently, the tight junction genes (*ZO‐1* and *Occludin*), along with L‐Trp levels in feces, serum, and brain tissues, were unaffected (Figure S6G−K). The transcriptional levels of the Sirt1/P53/P21/Rb pathway were assessed and found that EX‐527 significantly affected *Sirt1*, *P53*, *P21*, and *Rb* gene expression levels to varying extents (Figure S6L−O). Finally, the gut microbial composition was analyzed. Compared to the BP group, EX‐527 intervention resulted in decreased α‐diversity and OTUs, while no differences were observed in β‐diversity, with the LEFSe analysis revealing only five species differences (Figure S7A−D). At both the phylum and genus levels, varying degrees of effect on the species composition were noted, including a reduced relative abundance of beneficial microbiota (Figure S7E−I). Similarly, after EX‐527 intervention, the levels of *Bifidobacterium* significantly decreased (Figure S7J). Therefore, these results indicate that the effect of *B. pseudocatenulatum* NCU‐08 regulating the Sirt1/P53/P21/Rb signaling pathway to delay aging in SAMP8 mice is reduced after intervention with EX‐527.

## 3 Discussion

Aging is a universal issue faced by countries around the globe, characterized by a decline in physiological functions that increases the risk of various age‐related diseases and ultimately leads to mortality (Zhang, Wang, et al. 2023). In recent years, a wealth of research has indicated that regulating the gut microbiota has emerged as a promising antiaging strategy, demonstrating significant potential and prospects (Cai et al. 2024; Gao et al. 2023). However, while probiotic supplementation is a common method for modulating gut microbiota, there remains a notable lack of development in novel strains with antiaging properties, warranting further exploration. In this study, metagenomics and culturomics were utilized to identify new antiaging strains within the feces of centenarians from Jiangxi Province. Furthermore, the research aimed to investigate the antiaging effects and mechanisms of these strains at both animal and cellular levels, thus providing new data to support the development of innovative live microbial preparations for antiaging.

This study employed metagenomic technology to conduct an in‐depth analysis of the gut microbiota composition in centenarians from Jiangxi Province, China. The results revealed a significant predominance of *Bacteroides* in the microbial structure, while the abundance of potentially pathogenic bacteria was relatively low (Figure 1 and Figure S1). These findings align with previous research reports (Pang et al. 2023), suggesting a close association between gut microbiota balance and healthy aging. Particularly noteworthy was the marked enrichment of *Bifidobacterium*, especially *B. pseudocatenulatum* , a result consistent with multiple recent studies on the microbiomes of long‐lived populations (Wang et al. 2022), indicating that this bacterial species may play an important role in the healthy aging process.

To further investigate the functional characteristics of these microorganisms, we successfully isolated and cultured 1508 strains of gut microbes using culturomics techniques (Table S2). Notably, among these, the number of *B. pseudocatenulatum* (109 strains) held an absolute predominance within the *Bifidobacterium* genus. This discovery not only validated the metagenomic analysis results but also confirmed the special status of this bacterial species in the gut of long‐lived populations at the cultivable strain level. Through systematic in vitro screening, we identified a *B. pseudocatenulatum* strain, NCU‐08, which exhibited excellent probiotic properties, including growth vitality, acid tolerance, and bile salt resistance. This strain demonstrated probiotic capabilities similar to those of *Bifidobacterium animalis* CP‐9 reported by Tsai et al. (2022), providing an ideal research material for subsequent animal experiments to explore its antiaging mechanisms.

To investigate the antiaging effects of *B. pseudocatenulatum* NCU‐08, we used the senescence‐accelerated mouse model SAMP8 as an aging animal model. The experimental results showed that *B. pseudocatenulatum* NCU‐08 intervention significantly improved aging‐related behavioral deficits, alleviated hippocampal neuronal damage, and reduced the expression of the senescence marker β‐galactosidase. Additionally, NCU‐08 decreased the relative abundance of *Bacteroides* while increasing the relative abundance of the beneficial bacterium *Lactobacillus* (Figure 2). These findings are consistent with previous reports on the antiaging effects of probiotics (Fang et al. 2021), such as a probiotic combination that effectively improved behavioral performance in SAMP8 mice, suppressed neuroinflammation, upregulated Sirt1 expression to protect hippocampal neurons, and modulated gut microbiota. Therefore, *B. pseudocatenulatum* NCU‐08 effectively ameliorated aging characteristics in SAMP8 mice.

Currently, tryptophan metabolism has emerged as a potential target for antiaging interventions and has garnered widespread attention (Goot and Nollen 2013) By modulating the tryptophan metabolic pathway—particularly by inhibiting the kynurenine pathway and elevating NAD^+^ levels—it may be possible to achieve effective strategies for delaying aging (Braidy et al. 2011; Dang et al. 2023). Notably, targeted metabolomic analysis revealed that NCU‐08 significantly increased L‐Trp levels in aging mice (Figure 3), suggesting that L‐Trp may be the active compound responsible for NCU‐08's antiaging effects in SAMP8 mice. L‐Trp is a neutral aromatic amino acid containing an indole group and is an essential amino acid for humans and animals. It can alleviate anxiety, depression, and insomnia (Zhang et al. 2024), promote infant neurodevelopment (Manjarrez et al. 1998), and maintain gut health in hosts (Chen et al. 2024). In the field of aging research, L‐Trp has been shown to improve neurotransmission and memory behavior in aged rats (Esteban et al. 2010). A high‐L‐Trp diet can prevent age‐related declines in hippocampal serotonin levels, thereby enhancing cognitive function in aging individuals (Musumeci et al. 2017). Dietary L‐Trp supplementation also significantly ameliorated neurodegeneration and inflammatory responses in D‐galactose‐induced aging mice (Yin et al. 2021). Based on these findings, we hypothesized that L‐Trp might mitigate aging in SAMP8 mice. Further in vitro experiments confirmed this hypothesis: Compared to other tryptophan metabolites (XA and ILA), L‐Trp exhibited more pronounced cytoprotective effects, effectively alleviating D‐galactose‐induced cellular senescence (Figure S3). Interestingly, we also detected the presence of L‐Trp in the fermentation supernatant of NCU‐08 (Figure 3L), suggesting that NCU‐08 may produce L‐Trp either directly or through metabolic substrates. Notably, L‐Trp may be metabolized into other indole derivatives, such as indole‐3‐acetic acid, to exert antiaging effects. In summary, these findings provide evidence that NCU‐08 may delay aging by modulating L‐Trp levels.

To further investigate the antiaging effects of L‐Trp at the animal level, we administered L‐Trp interventions to aging SAMP8 mice. The results demonstrated that, similar to the previously observed effects of NCU‐08 strain intervention (Figure 2), L‐Trp intervention alone significantly improved motor dysfunction in aging mice, alleviated hippocampal neuronal damage, and reduced the expression levels of the aging marker β‐gal (Figure 4). This finding aligns with the results reported by He et al. (2020), suggesting that L‐Trp possesses anti‐aging properties. Yu et al. found that *Lactobacillus plantarum* FLPL05 altered the composition of the gut microbiota in naturally aging mice, particularly the proportion of *Lactobacillus* (Yu et al. 2023). As a dominant gut microbiota in mice, *Lactobacillus* has been proven to delay aging by modulating gut microbiota balance and oxidative stress levels (Chen et al. 2023, 2022). Similarly, our results support this notion, as L‐Trp modified the gut microbiota composition in SAMP8 mice, notably increasing the relative abundance of *Lactobacillus*, while no significant change was observed in *Bifidobacterium* abundance. Additionally, studies have shown that Li et al. discovered theaflavins in black tea alleviated aging‐related cognitive dysfunction via the microbiota‐gut‐brain axis (Li, Zhang, et al. 2023). Therefore, we hypothesized whether L‐Trp exerts its antiaging effects. Our results revealed that L‐Trp intervention significantly elevated L‐Trp levels in feces, serum, and brain tissues, while also markedly increasing the gene expression of tight junction proteins ZO‐1 and Occludin in intestinal tissues (Figure 4). However, our findings only suggest that L‐Trp may regulate brain aging, and further precise experiments are required for validation.

To further investigate the antiaging mechanism of L‐Trp, we established a D‐galactose‐induced cellular senescence model using HT22 cells. The experimental results demonstrated that compared with the D‐gal group, L‐Trp treatment significantly enhanced cell viability (*p* < 0.01) and reduced the expression level of senescence marker β‐gal (*p* < 0.05), confirming the definitive antiaging effect of L‐Trp at the cellular level (Figure 5). Additionally, to identify the target genes of L‐Trp, we examined the expression of several common senescence‐related genes using RT‐qPCR. Compared with the D‐gal group, only two genes showed significant changes after L‐Trp intervention: a marked decrease in P53 expression and a significant increase in Sirt1 expression. These findings are consistent with numerous studies highlighting the importance of Sirt1 in aging. For instance, Sirt1, an NAD^+^‐dependent deacetylase, has been shown to extend lifespan and improve health status in mice (Mitchell et al. 2014). Furthermore, resveratrol has been reported to promote longevity and enhance glucose homeostasis by activating NAD^+^‐dependent Sirt1 (Koo and Montminy 2006). At the molecular level, Sirt1 can inhibit cellular senescence by deacetylating P53 (Cheng et al. 2021). Additionally, studies have demonstrated that Serpine1 promotes senescence in type II alveolar epithelial cells through the P53‐P21‐Rb signaling axis (Jiang et al. 2017). Based on this evidence, we hypothesized that L‐Trp might exert its antiaging effects by promoting NAD^+^ biosynthesis through the kynurenine pathway, thereby activating Sirt1 expression and ultimately inhibiting the P53/P21/Rb signaling pathway. To test this hypothesis, we systematically analyzed key molecules in the tryptophan metabolic pathway, NAD^+^ levels, and the Sirt1/P53/P21/Rb signaling pathway. Fortunately, our results confirmed this hypothesis. The experimental data revealed that compared with the D‐gal group, L‐Trp treatment significantly enhanced the activity of metabolic enzymes (IDO, TDO, KMO, and NADS) and increased NAD^+^ content, while markedly altering the expression of key proteins (Sirt1, P53, P21, and Rb). These results collectively suggest that L‐Trp may exert its antiaging effects by promoting NAD^+^ biosynthesis and subsequently regulating the Sirt1/P53/P21/Rb signaling pathway.

Finally, to validate the role of the Sirt1/P53/P21/Rb signaling pathway in aging, we employed the inhibitor EX‐527 in both in vitro and in vivo experiments. EX‐527 is a potent and selective Sirt1 inhibitor that has been widely used to investigate aging mechanisms (Wan et al. 2021; Xing et al. 2023). Previous studies have demonstrated that isoliquiritigenin prevents doxorubicin‐induced liver injury in rats by upregulating and activating SIRT1, while EX‐527 reverses this protective effect (Al‐Qahtani et al. 2022). Additionally, EX‐527 reduced the NAD^+^/NADH ratio, increased acetylated p53 levels, abolished the cardioprotective effects of taurine in transverse aortic constriction mice, and enhanced apoptosis and oxidative stress (Liu et al. 2020). Similarly, our data confirm the efficacy of EX‐527 in inhibiting Sirt1. Both cellular and animal experiments demonstrated that EX‐527 suppresses Sirt1 gene expression and inhibits the activation of the Sirt1/P53/P21/Rb signaling pathway (Figure 6). Notably, EX‐527 does not affect tryptophan metabolism‐related indicators (the enzyme activities of IDO, TDO, KMO, NADS, and NAD^+^ levels) and fails to ameliorate hippocampal neuronal damage, suggesting that the neuroprotective role of this pathway may be tissue‐specific. These results confirm that strain NCU‐08 delays aging by modulating L‐Trp to activate the Sirt1/P53/P21/Rb pathway.

Here, there are still some limitations to this study. (1) First, due to the limited number of centenarians recruited for this study, future research should include a larger sample size. Additionally, the relative abundance of *B. pseudocatenulatum* should be assessed in different age groups to confirm its high enrichment in the guts of centenarians. (2) Due to the requirements for large sample sizes, complex indicators, and long‐term observation when evaluating multi‐organ systems, this study did not conduct comprehensive anti‐aging research on NCU‐08's effects on other organs, thus preventing a full assessment of its antiaging potential. (3) This study did not precisely quantify the colonization of *B. pseudocatenulatum* in mice. Further validation using digital PCR or metagenomics approaches is required for accurate measurement. (4) During the process of NCU‐08 intervention on the gut microbiota, the tryptophan synthesized by the gut microbiota may influence the results, and further studies using germ‐free animals are needed to eliminate this factor. Meanwhile, it remains unclear whether NCU‐08 produces L‐Trp through synthesis or substrate utilization. Further investigation based on genomic data is required to elucidate the specific metabolic pathway. (5) Since EX‐527 has not completely silence the gene, future studies should employ gene knockout techniques to validate the role of the Sirt1 gene.

## 4 Conclusion

**FIGURE 7:**
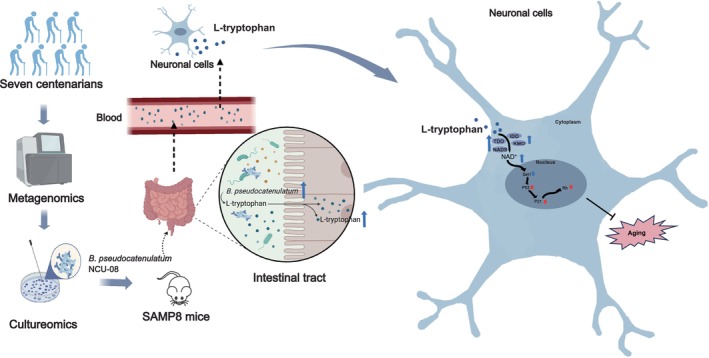
Potential molecular mechanism of *B. pseudocatenulatum* NCU‐08 in delaying aging of SAMP8 mice by regulating the Sirt1/P53/P21/Rb signaling pathway. *B. pseudocatenulatum* NCU‐08 was isolated from the feces of seven centenarians using metagenomics. After gavage administration to SAMP8 mice, the strain added the content of L‐tryptophan. L‐tryptophan traversed the intestinal tract and blood–brain barrier, entered hippocampal neurons, and was metabolized to produce NAD^+^. The increased NAD^+^ activated the *Sirt1* gene, inhibiting the production of senescence‐associated proteins P53, P21, and Rb, which decreased the production of senescence proteins, thereby delaying aging.

In this study, metagenomics and culturomics were used to reveal the compositional characteristics of the gut microbiota in centenarians. At the same time, a strain of *B. pseudocatenulatum* NCU‐08 with good probiotic properties was obtained based on the screening of probiotic properties. Furthermore, *B. pseudocatenulatum* NCU‐08 was confirmed at the animal level to significantly improve the behavioral characteristics of mice, reduce the expression of the senescence marker protein β‐gal, enhance the intestinal barrier, and alter the composition of the gut microbiota. At the same time, L‐Trp was confirmed to be the core molecule of the antiaging effect of *B. pseudocatenulatum* NCU‐08. In addition, combined with animal and cellular senescence models and inhibitor interventions, *B. pseudocatenulatum* NCU‐08 produced L‐Trp, which was converted into NAD^+^ through the kynurenine pathway, thereby activating the Sirt1/P53/P21/Rb signaling pathway to improve aging (Figure 7). Future research should try to discover other new strains in centenarian feces that may be equally important for aging and longevity. Furthermore, the genetic modification of *B. pseudocatenulatum* using synthetic biology techniques may open new avenues for anti‐aging research. This study provides important data support for the development of antiaging microbial agents.

## 5 Materials and Methods

### 5.1 Collection of Fecal Samples From Centenarians in Jiangxi Province, China

This study was collaboratively conducted by our team and Jiangxi Shanjian Biotechnology Co. Ltd. in Zhanggong District, Ganzhou City, Jiangxi Province, China, in 2019. It received approval from the Zhanggong District Medical Ethics Committee (Approval No: 2019001), and all research procedures adhered strictly to the committee's ethical guidelines. Prior to recruitment, all subjects were thoroughly informed about the study's processes and significance. They voluntarily provided fecal samples and signed a written informed consent form. Fresh fecal samples were collected from each participant using sterile swabs, which were placed in sterile 50 mL tubes. Each participant was required to submit two samples: one without glycerol and another containing 30% glycerol, both clearly labeled. Following collection, all fecal samples were immediately stored on dry ice. Upon arrival at the laboratory, the samples were then stored at −80°C until further analysis.

### 5.2 Analysis of Metagenomics and Culturomics

Total DNA was extracted from the feces of centenarians (without adding 30% glycerol) using a fecal genomic DNA extraction kit (DP328‐02, 50 times, Tiangen, China) and stored at −20°C until whole genome shotgun (WGS) sequencing was performed. The sequencing was carried out on the Illumina NovaSeq/HiSeq sequencing platform at Personal Biotechnology Co. Ltd. in Shanghai, China. Following the methods from previous literature (Gu et al. 2021), the obtained raw data underwent quality assessment and filtering, after which the processed data were used for analysis. QIIME software was utilized to determine the composition and abundance of each sample at the phylum, class, order, family, genus, and species levels. Subsequently, R 3.5.1 software was employed to perform cluster analysis of the top 50 relative abundance operational taxonomic units that exhibited significant differences between groups, and a heatmap was generated. All raw data files can be accessed from the National Center for Biotechnology Information (NCBI) online database, and the specific GenBank accession number can be found in the relevant record (PRJNA1209849).

In this study, feces containing 30% glycerol from centenarians were extracted from a −80°C freezer, and the fecal microorganisms were isolated based on previous research (Bazireh et al. 2020; Kaya et al. 2022). The specific steps included using five common media for microbial cultivation, including Luria‐Bertani (LB) (HB9305, 250 g, HOPEBIO, China), Brain‐Heart Infusion (BHI) (HB8478, 250 g, HOPEBIO, China), DeMan, Rogosa and Sharpe (MRS) (HB0384, 250 g, HOPEBIO, China), Trypticase Soy Broth (TSB) (HB4114, 250 g, HOPEBIO, China), and BBL Agar Medium (HB0396, 250 g, HOPEBIO, China). After cultivating for 48 h, single colonies were picked and transferred into liquid culture medium for further growth. Subsequently, DNA was extracted and sent to Sangon Biotech (Shanghai) Co. Ltd. for 16S rRNA sequencing. Primers were 27F (5′−GCAGAGTTCTCGGAGTCACGAAGAGTTTGATCCTGGCTCAG−3′) and 1492R (5′−AGCGGATCACTTCACACAGGACTACGGCTACCTTGTTACGA−3′). Finally, the nucleotide BLAST analysis was used in the NCBI database (https://blast.ncbi.nlm.nih.gov/Blast.cgi). In addition, a Neighbor‐Joining phylogenetic tree was constructed for the isolated strain using MEGA 6.0 software (Myung et al. 2014), combining morphological characteristics (colony morphology and gram staining) and phylogenetic analysis to determine the taxonomic status of the strain. Furthermore, based on previously reported methods (Li, He, et al. 2023; Othman and Karim 2023), microbial characteristics of the strain were evaluated, including growth curve, acid resistance, bile salt resistance, acid production, salt tolerance (NaCl), and high‐temperature resistance.

### 5.3 Experimental Animals and Treatments

The experimental animals used in this study were 5‐month‐old male SAMP8 mice, a model of accelerated aging, and SAMR1 mice, a model of normal aging, purchased from SPF (Beijing) Biotechnology Co. Ltd. Prior to the experiment, the mice were acclimatized for 1 week under standard Specific Pathogen‐Free (SPF) conditions, which included a 12‐h light/dark cycle, a temperature of 22°C ± 3°C, and a relative humidity of 50% ± 15%. Mice were also provided with free access to food (Standard maintenance diet for mice/rats, SPF‐F02‐001, SPF (Beijing) Biotechnology Co. Ltd.) and water. L‐Trp (ST8320, 200 mg, Solarbio, China) and the inhibitor Selisitat (EX‐527) (HY‐15452, 50 mg, MedChemExpress, USA) were dissolved in sterile dimethyl sulfoxide (DMSO) (D8370, 500 mL, Solarbio, China). Furthermore, *B. pseudocatenulatum* NCU‐08 was inoculated into MRS broth containing cysteine. After 48 h of growth, the culture was centrifuged to obtain the precipitate, which was then resuspended in gelatin saline to achieve a bacterial suspension with a concentration of 10^9^ CFU/mL. The doses of L‐Trp and EX‐527 were selected based on previously published literature (Liu et al. 2020; Zhang, Hu, et al. 2023) and preliminary experimental results.

To investigate the antiaging effect of *B. pseudocatenulatum* NCU‐08 derived from centenarians on SAMP8 mice, the mice were divided into three groups: (1) Control group (C group, *n* = 10), SAMR1 mice were gavaged daily with an equivalent volume of gelatin saline as the probiotics for 12 weeks; (2) Model group (M group, *n* = 10), SAMP8 mice were gavaged daily with an equivalent volume of gelatin saline as the probiotics for 12 weeks; (3) *B. pseudocatenulatum* NCU‐08 group (BP group, *n* = 10), SAMP8 mice were gavaged daily with 10^9^ CFU/mL of *B. pseudocatenulatum* NCU‐08 for 12 weeks. One week after the treatment ended, all mice underwent behavioral assessments and fecal sample collection. Finally, the mice were anesthetized, and serum, brain, and colon samples were collected. Two brain tissue samples per group were fixed with 4% paraformaldehyde for hematoxylin–eosin (H&E) staining, while the remaining samples were stored in a −80°C freezer for subsequent research.

To explore the antiaging effect of L‐Trp on SAMP8 mice, the mice were divided into three groups: (1) Model group (M group, *n* = 10), SAMP8 mice were gavaged daily with an equivalent volume of gelatin saline as the probiotics for 12 weeks; (2) *B. pseudocatenulatum* NCU‐08 group (BP group, *n* = 10), SAMP8 mice were gavaged daily with 10^9^ CFU/mL of *B. pseudocatenulatum* NCU‐08 for 12 weeks; (3) L‐tryptophan group (L‐Trp group, *n* = 10), SAMP8 mice were gavaged daily with 20 mg/kg of L‐Trp for 12 weeks. One week after the treatment ended, the mice were tested for relevant indicators as described above.

To verify the antiaging mechanism of *B. pseudocatenulatum* NCU‐08, the EX‐527 inhibitor was selected for intervention. The mice were divided into three groups: (1) Model group (M group, *n* = 10), SAMP8 mice were gavaged daily with an equivalent volume of gelatin saline as the probiotics for 12 weeks; (2) *B. pseudocatenulatum* NCU‐08 group (BP group, *n* = 10), SAMP8 mice were gavaged daily with 10^9^ CFU/mL of *B. pseudocatenulatum* NCU‐08 for 12 weeks; (3) *B. pseudocatenulatum* NCU‐08 + EX‐527 group (BP + EX‐527 group, *n* = 10), SAMP8 mice were gavaged daily with 10^9^ CFU/mL of *B. pseudocatenulatum* NCU‐08 and received intraperitoneal injections of 5 mg/kg of the inhibitor EX‐527, which was administered 4 h before the probiotic intervention, for 12 weeks. One week after the treatment ended, the mice were tested for relevant indicators as described above.

All animal experiments were conducted in accordance with the Guidelines for the Care and Use of Laboratory Animals from Nanchang University and were approved by the Animal Ethics Committee of Nanchang University (Ethics Number: NCULAE‐20221228051).

### 5.4 Behavioral Experiments

Based on previous research methods (Wang et al. 2023), a series of behavioral experiments were conducted, including the hanging wire test, open field test, and pole test. In brief, the hanging wire test recorded the time it took for the mice to descend from the top of the rod to the bottom. The open field test measured the total distance traveled by the mice and the distance traveled in the central area in 5 min. The pole test recorded the duration the mice held onto a rope with their forepaws. Each experiment involved three parallel tests for each mouse, and the average was taken as the result.

### 5.5 H&E Staining

Mice brain tissue was fixed in 4% paraformaldehyde and subsequently dehydrated and embedded. Tissue samples were then sliced into 5‐micron thick sections using a microtome (CM1850, Leica, Germany). The sections were stained with H&E and observed for morphological changes using an optical microscope (Eclipse 80i, Nikon, Japan).

### 5.6 Cell Models and Interventions

HT22 mouse hippocampal neuronal cells were purchased from Wuhan Pricella Biotechnology Co. Ltd. (CL‐0697, Pricella, China) and cultured in plates with DMEM medium (11995, 500 mL, Solarbio, China) supplemented with 10% Fetal Bovine Serum (FBS) (S9030, 100 mL, Solarbio, China) and 1% penicillin–streptomycin (P1400, 100 mL, Solarbio, China). The cells were maintained at 37°C in a 5% CO_2_ atmosphere.

In order to investigate the antiaging effects of L‐Trp at the cellular level, an aging cell model using D‐gal (SG8010, 100 mg, Solarbio, China) was established. The experiment was divided into three groups: (1) PBS group, which received an intervention of an equal volume of PBS for 24 h; (2) D‐gal group, which received an intervention of 100 mM D‐gal for 24 h; (3) D‐gal + L‐Trp group, which received an intervention of 100 mM D‐gal and 20 μM L‐Trp for 24 h.

To explore the mechanism of L‐Trp's antiaging effect, the EX‐527 was selected for verification. The experiment was divided into three groups: (1) D‐gal group, which received an intervention of 100 mM D‐gal for 24 h; (2) D‐gal + L‐Trp group, which received an intervention of 100 mM D‐gal and 20 μM L‐Trp for 24 h; (3) D‐gal + L‐Trp + EX‐527 group, which received an intervention of 100 mM D‐gal, 20 μM L‐Trp, and 10 μM EX‐527 for 24 h.

HT22 cells were treated once they reached approximately 70% confluence. After all interventions were completed, cell samples were collected for subsequent experiments.

### 5.7 Cell Viability Assay

Cell viability for the treated groups was assessed strictly following the protocol provided with the Cell Counting Kit‐8 (CCK‐8) (C0038, 500 times, Biyotime China).

### 5.8 Western Blot

Brain tissue and cell pellets were lysed in RIPA lysis buffer containing a proteinase inhibitor mixture (50 × Cocktail) (G2006‐250UL, 250 μL, Servicebio, China). The samples were homogenized on ice and centrifuged at 10,000 rpm for 4°C. The collected supernatant was used to obtain protein samples. These protein samples were separated by sodium dodecyl sulfate‐polyacrylamide gel electrophoresis (SDS‐PAGE). After separation, proteins were transferred to a polyvinylidene fluoride (PVDF) membrane, which was subsequently blocked with nonfat milk and incubated overnight at 4°C with primary antibodies. The primary antibodies included Beta Galactosidase Monoclonal antibody (β‐gal) (66586‐1‐Ig, 50 μL, Proteintech, China), SIRT1 Rabbit pAb (Sirt1) (YT4302, 40 μL, Immunoway, USA), p53 (6C4) Mouse mAb (P53) (YM3052, 40 μL, Immunoway, USA), Rb Rabbit pAb (Rb) (YT4023, 40 μL, Immunoway, USA), p21 Rabbit mAb (P21) (R381102, 50 μL, Zenbio, China), and Beta Actin Monoclonal antibody (β‐actin) (66009‐1‐Ig, 100 μL, Proteintech, China). Subsequently, the membranes were incubated with corresponding secondary antibodies (anti‐mouse, SA00001‐1, 100 μL, Proteintech, China; anti‐rabbit, SA00001‐2, 100 μL, Proteintech, China). Finally, a chemiluminescent solution (32209, 250 mL, Thermo Fisher, USA) was added to detect the specific proteins. Protein bands were quantified using ImageJ software (v1.8.0, USA) for subsequent analysis.

### 5.9 RT‐qPCR

Total RNA from brain tissue, colon, and cell pellets was extracted using TRIzol reagent (10296010CN, 100 mL, Gibco, USA). Subsequently, RNA was reverse transcribed to cDNA using a reverse transcription kit (RR047A, Takara, Japan). Following this, real‐time reverse transcriptase quantitative polymerase chain reaction (RT‐qPCR) was performed on a ViiA 7 Real‐Time PCR System (Applied Biosystems, USA) using the TB Green Premix Ex TaqTM (with Tli RNaseH Plus) kit (RR420A, Takara, Japan) to quantify the relative expression of specific genes in the samples. The genes analyzed included *P16*, *P21*, *CD38*, *Klotho*, Silent information regulator 1 (*Sirt1*), Silent information regulator 3 (*Sirt3*), Silent information regulator 6 (*Sirt6*), Activating Transcription Factor 4 (*ATF4*), *P53*, *Occludin*, and Zonula Occludens‐1 (*ZO‐1*). Results were normalized to the housekeeping gene glyceraldehyde‐3‐phosphate dehydrogenase (*GAPDH*). Additionally, total DNA from mouse feces was extracted using a fecal genomic DNA extraction kit (DP328‐02, 50 times, Tiangen, China), and similar fluorescence quantification was conducted to determine the relative levels of *Bifidobacterium* (*Bif*) in the feces. Results were normalized to the bacterial reference gene 16S rDNA (*16S*). Relative expression levels were calculated using the 2^−ΔΔCt^ method, with each experiment conducted in six parallel replicates. The primer sequences were shown in Table 1.

**TABLE 1:** Primer sequences for quantitative RT‐qPCR.

Primers	Forward (5′−3′)	Reverse (5′−3′)
P16	CGTACCCCGATTCAGGTGAT	TTGAGCAGAAGAGCTGCTACG
P21	AGAGGGAGCCTGAAGACTGT	TCAGACACCAGAGTGCAAGA
CD38	TGGTCCTGATCGCCTTGGTAGTAG	GTGTCCTCCAGGGTGAACATCTTTC
Klotho	TGTGAATGAGGCTCTGAAAGC	GAGCGATCACTAAGTGAATACG
Sirt1	GGAGCAGATTAGTAAGCGGCTTG	GTTACTGCCACAGGAACTAGAGG
Sirt3	GCCCAATGTCACTCACTACTTCCTG	CCACCAGCCTTTCCACACCATG
Sirt6	GCACCGTGGCTAAGGCAAGG	GTGATGGACAGGTCGGCGTTC
ATF4	CCTATAAAGGCTTGCGGCCA	TGAAGAGCGCCATGGCTTAG
Rb	GATAACCTTGAACCTGCTTGTC	GGAGATATGCTAGACGGTACAC
P53	CACAGCGTGGTGGTACCTTATGAG	TGGTAAGGATAGGTCGGCGGTTC
ZO‐1	TTTTTGACAGGGGGAGTGG	TGCTGCAGAGGTCAAAGTTCAAG
Occludin	ATGTCCGGCCGATGCTCTC	TTTGGCTGCTCTTGGGTCTGTAT
β‐galactosidase	CGGATACCCCGCTTCTACTG	AGTTCCAGGGCACGTACATC
GAPDH	CTCATGACCACAGTCCATGC	CACATTGGGGGTAGGAACAC
Bif	TCGCGTC(C/T)GGTGTGAAAG	CCACATCCAGC(A/G)TCCAC
16S	ACTCCTACGGGAGGCAGCAGT	TATTACCGCGGCTGCTGGC

### 5.10 ELISA Analysis

The mouse tryptophan ELISA kit (MM‐0756M1, 96T, MEIMIAN, China) was used to measure the L‐Trp levels in bacterial fermentation supernatants, serum, brain tissue, and feces, following the manufacturer's instructions. Additionally, ELISA kits were employed to assess the enzymes and products associated with the tryptophan metabolic pathway in brain tissue and cell samples, including indoleamine‐2,3‐dioxygenase (IDO) (MM‐0641M1, 96T, MEIMIAN, China), tryptophan‐2,3‐dioxygenase (TDO) (MM‐46550M1, 96T, MEIMIAN, China), kynurenine‐3‐monooxygenase (KMO) (MM‐46566M1, 96T, MEIMIAN, China), NAD synthetase (NADS) (MM‐48073M1, 96T, MEIMIAN, China), and nicotinamide adenine dinucleotide (NAD^+^) (MM‐1010M1, 96T, MEIMIAN, China).

### 5.11 High‐Throughput Sequencing

Microbial genomic DNA from mice feces was extracted using a DNA extraction kit (DP712‐1, 50 preps, Tiangen, China) according to the manufacturer's protocol. The concentration and quality of the purified DNA were measured with a spectrophotometer (NanoDrop, Thermo Scientific). Subsequently, the V4 region of the 16S rDNA gene in each sample was amplified using primers 515F/806R (515F was 5′−GCACCTAAYTGGGYDTAAAGNG−3′ and 806R was 5′−TACNVGGGTATCTAATCC−3′), and sequencing was performed on the Illumina NovaSeq platform. The DADA2 method was then used to generate ASV/OTU feature sequences. The ASV/OTU‐based approach allows for the analysis of the composition and structure of the microbiota. All raw data files can be accessed from the NCBI online database, and the specific GenBank accession number can be found in the relevant record (PRJNA1209808, PRJNA1209822, and PRJNA1209832) (https://www.ncbi.nlm.nih.gov/).

### 5.12 Analysis of Tryptophan Metabolism

Fresh feces of mice were collected and sent to Shanghai Personal Biotechnology Co. Ltd. for targeted metabolomics analysis of 31 substances related to tryptophan metabolism. In brief, 500 μL of methanol was added to a 1.5 mL centrifuge tube containing fecal samples, which were then vortexed and centrifuged for 10 min at 4°C at 12,000 rpm. The supernatant was collected for analysis. Subsequently, ultra‐performance liquid chromatography (UPLC) (ExionLC AD, https://sciex.com.cn/) and tandem mass spectrometry (MS/MS) (QTRAP6500+, https://sciex.com.cn/) were used for detection. Total ion chromatograms of mass spectral peaks were generated by controlling the software for data acquisition. A database was constructed based on standard compounds for qualitative analysis of the mass spectrometry data. For metabolite quantification, the multiple reaction monitoring (MRM) mode of the triple quadrupole mass spectrometer was employed. Peak areas of the extracted ion chromatograms for all metabolites were integrated, and area corrections were applied for the same metabolites across different samples. Data acquisition was performed using Analyst 1.6.3, and quantification was done using MultiQuant 3.0.3. The data obtained were analyzed using R 3.5.1 software for principal component analysis (PCA) and clustering heatmap analysis.

### 5.13 Correlation Analysis

According to previous research (Jiang et al. 2022), a correlation heatmap analysis of mice aging characteristics, gut microbiota, and metabolic products was performed using the Correlation Plot in Origin 2021 software.

### 5.14 Statistical Analysis

Data analysis was conducted using GraphPad Prism 9.0 software (GraphPad Software, San Diego, CA, USA). Experimental data were initially analyzed using one‐way ANOVA for statistical significance, followed by Tukey's test for multiple comparisons. The Shapiro–Wilk test was used to determine data normality. The statistical method used for the correlation heatmap was the Pearson correlation test. Results are presented as mean ± standard deviation (SD) or median (interquartile range), with *p* < 0.05 considered statistically significant.

## Author Contributions

T.X. contributed to methodology, investigation, formal analysis, visualization, and writing – original draft. X.W. participated in methodology. Y.Z. and Y.C. participated in visualization. X.Z. and Q.Z. contributed to investigation. J.L. and J.W. took part in supervision. T.C. contributed to conceptualization and writing – review and editing.

## Ethics Statement

This study involving the collection of fresh fecal samples from centenarians was approved by the Zhanggong District Medical Ethics Committee (Approval Number: 2019001). All research procedures were conducted in strict compliance with the committee's ethical guidelines, with informed consent obtained from all centenarian participants. Additionally, animal experiments were approved by the Nanchang University Animal Ethics Committee (Ethics Number: NCULAE‐20221228051) and performed in strict adherence to the Guidelines for the Care and Use of Laboratory Animals established by Nanchang University.

## Conflicts of Interest

The authors declare no conflicts of interest.

## Supporting information


Appendix S1.

## Data Availability

The raw data from the 16S rRNA sequencing generated in this study has been deposited in the NCBI database (PRJNA1209849, PRJNA1209808, PRJNA1209822, and PRJNA1209832).
