# Supplementary material for: L‐Tryptophan Produced by Bifidobacterium pseudocatenulatum NCU‐08 Delays Aging in SAMP8 Mice by Activating the Sirt1/P53/P21/Rb Signaling Pathway
Source: Aging Cell. 2025 Jul 11;24(9):e70166. doi: 10.1111/acel.70166 (PMC12419854; doi:10.1111/acel.70166)
Supplement: Supplementary file 1 — Appendix S1. [file ACEL-24-e70166-s001.docx]

**Supplementary Materials**

**L-Tryptophan produced by *Bifidobacterium pseudocatenulatum* NCU-08 delays aging in SAMP8 mice by activating the** **Sirt1/P53/P21/Rb signaling pathway**

Tangchang Xu^1^, Xiaoyun Wu^2^, Yifei Zhang^3^, Yujie Cai^3^, Xinfeng Zhang^3^, Qingwei Zeng^3^, Jie Luo^4^, Jing Wei^5^ & Tingtao Chen^1,3,5*^

^1^School of Life Sciences, Nanchang University, Nanchang 330031, China.

^2^Second College of Clinical Medicine, Jiangxi Medical College, Nanchang University, Nanchang 330031, China.

^3^Jiangxi Province Key Laboratory of Bioengineering Drugs, School of Pharmacy, Jiangxi Medical College, Nanchang University, Nanchang 330031, China.

^4^School of Public Health, Jiangxi Medical College, Nanchang University, Nanchang 330031, China.

^5^China National Engineering Research Center for Bioengineering Drugs and the Technologies, Institute of Translational Medicine, Jiangxi Medical College, Nanchang University, Nanchang 330031, China.

*Correspondence: chentingtao1984@163.com or chentingtao@ncu.edu.cn (Tingtao Chen)

**Table S1 Collecting information from seven centenarians in 2019**

| Number | Sample | Sex | Age | Height (m) | Weight (kg) | Body Mass Index (BMI) |
| --- | --- | --- | --- | --- | --- | --- |
| 1 | Fb1 | Female | 100 | 1.56 | 42.5 | 17.46 |
| 2 | Fb2 | Female | 107 | 1.54 | 46.3 | 19.52 |
| 3 | Fb3 | Female | 101 | 1.62 | 45.7 | 17.41 |
| 4 | Fb4 | Female | 104 | 1.52 | 43.5 | 18.83 |
| 5 | Fb5 | Female | 101 | 1.60 | 42.8 | 16.72 |
| 6 | Fb6 | Female | 102 | 1.54 | 47.1 | 19.86 |
| 7 | Fb7 | Female | 101 | 1.50 | 40.1 | 17.82 |

Note: Fb1: Fbkuang; Fb2: Fblai; Fb3: Fblei; Fb4: Fblin; Fb5: Fbliu; Fb6: Fbwang; Fb7: Fbzhao.


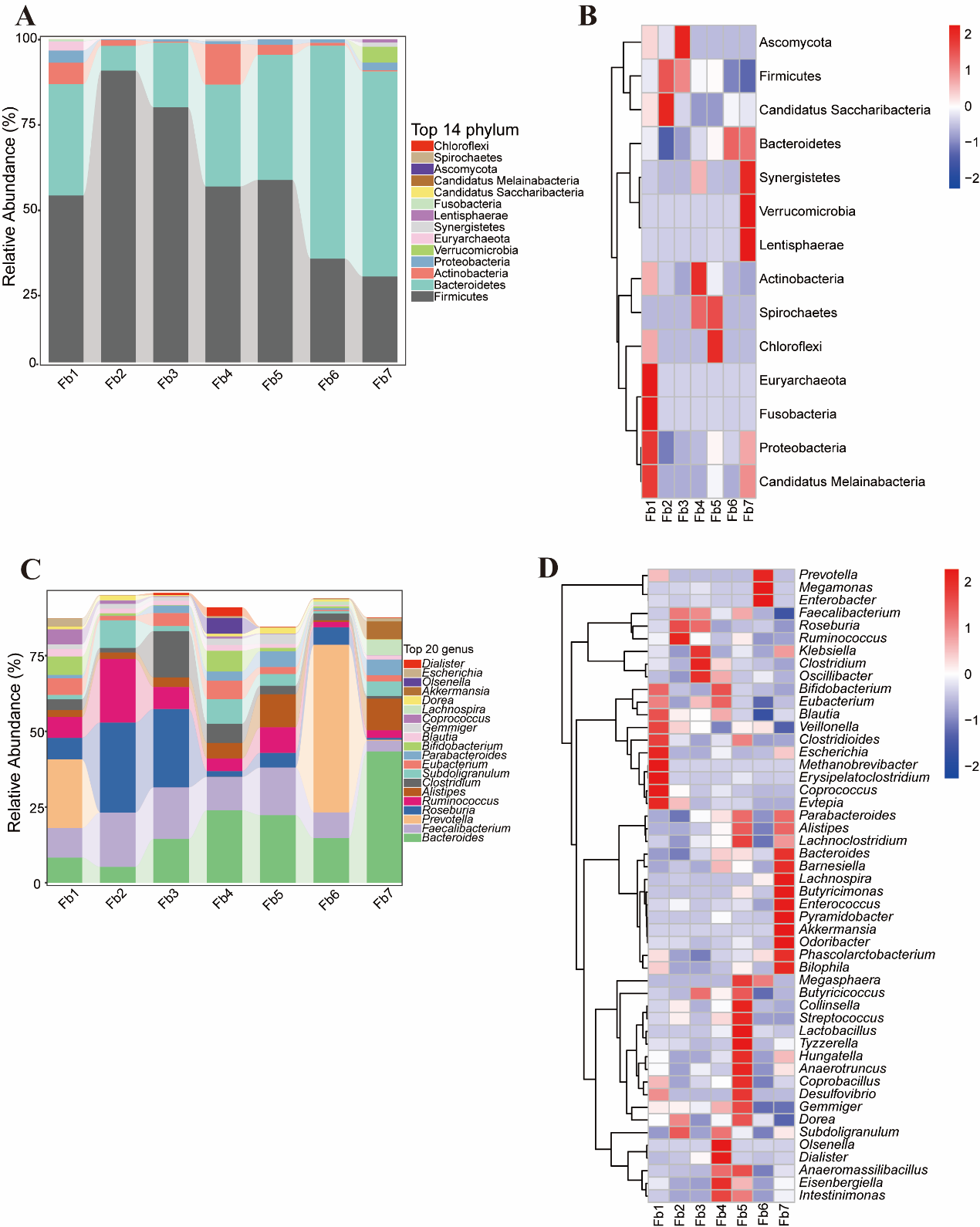


**Fig. S1 Characteristics of fecal microbiota at phylum and genus levels in seven centenarians.** (A) Taxonomic composition of fecal microbiota at the phylum level. (B) Heatmap of clustering for differential species at phylum level. (C) Taxonomic composition of fecal microbiota at the genus level. (D) Heatmap of clustering for differential species at genus level. Fb1: Fbkuang; Fb2: Fblai; Fb3: Fblei; Fb4: Fblin; Fb5: Fbliu; Fb6: Fbwang; Fb7: Fbzhao.

**Table S2 Characteristics of fecal microbial isolation in seven centenarians**

| **No.** | **Genus** | **Species** | **Fb1** | **Fb2** | **Fb3** | **Fb4** | **Fb5** | **Fb6** | **Fb7** |
| --- | --- | --- | --- | --- | --- | --- | --- | --- | --- |
| 1 | *Clostridium* | *Clostridium butyricum* | -^a^ | -^a^ | 15 | 2 | -^a^ | -^a^ | 1 |
| 2 |  | *Clostridium perfringens* | -^a^ | 1 | 2 | 4 | -^a^ | -^a^ | 1 |
| 3 | *Enterococcus* | *Enterococcus raffinosus* | 8 | -^a^ | -^a^ | -^a^ | -^a^ | -^a^ | -^a^ |
| 4 |  | *Enterococcus lactis* | 13 | 5 | 2 | 9 | 18 | 4 | 2 |
| 5 |  | *Enterococcus faecalis* | 8 | 15 | 16 | 24 | 5 | 6 | 7 |
| 6 |  | *Enterococcus avium* | -^a^ | 12 | -^a^ | 8 | -^a^ | -^a^ | 1 |
| 7 | *Lactiplantibacillus* | *Lactiplantibacillus plantarum* | 7 | -^a^ | 12 | 24 | 3 | -^a^ | -^a^ |
| 8 | *Limosilactobacillus* | *Limosilactobacillus reuteri* | 1 | 3 | -^a^ | -^a^ | 32 | 9 | 10 |
| 9 | *Ligilactobacillus* | *Ligilactobacillus salivarius* | 18 | 6 | 21 | 8 | 34 | -^a^ | 2 |
| 10 | *Lactobacillus* | *Lactobacillus paragasseri* | -^a^ | -^a^ | -^a^ | -^a^ | 19 | 3 | 1 |
| 11 |  | *Lactobacillus fermentum* | 16 | 2 | 25 | 8 | 43 | 5 | 15 |
| 12 |  | *Lactobacillus mucosae* | 12 | 3 | 9 | 31 | 12 | 25 | -^a^ |
| 13 |  | *Lactobacillus gasseri* | 15 | 6 | 24 | 36 | -^a^ | 1 | -^a^ |
| 14 | *Lacticaseibacillus* | *Lacticaseibacillus paracasei* | -^a^ | -^a^ | 7 | -^a^ | 53 | -^a^ | -^a^ |
| 15 |  | *Lacticaseibacillus casei* | -^a^ | 9 | 4 | -^a^ | 2 | -^a^ | -^a^ |
| 16 | *Bacillus* | *Bacillus amyloliquefaciens* | -^a^ | -^a^ | 13 | -^a^ | -^a^ | 17 | 19 |
| 17 |  | *Bacillus cereus* | 2 | 8 | -^a^ | 12 | 2 | 18 | -^a^ |
| 18 | *Aeromonas* | *Aeromonas caviae* | -^a^ | 11 | -^a^ | -^a^ | -^a^ | -^a^ | -^a^ |
| 19 | *Citrobacter* | *Citrobacter freundii* | 6 | -^a^ | -^a^ | -^a^ | -^a^ | -^a^ | -^a^ |
| 20 | *Paraclostridium* | *Paraclostridium bifermentans* | -^a^ | -^a^ | 25 | -^a^ | -^a^ | -^a^ | -^a^ |
| 21 | *Salmonella* | *Salmonella enterica* | 6 | -^a^ | -^a^ | -^a^ | -^a^ | -^a^ | -^a^ |
| 22 | *Streptococcus* | *Streptococcus lutetiensis* | -^a^ | -^a^ | -^a^ | -^a^ | -^a^ | -^a^ | 2 |
| 23 |  | *Streptococcus anginosus* | -^a^ | 18 | -^a^ | -^a^ | -^a^ | -^a^ | -^a^ |
| 24 | *Bifidobacterium* | *Bifidobacterium longum* subsp. *longum* | 5 | -^a^ | 1 | 16 | -^a^ | -^a^ | -^a^ |
| 25 |  | *Bifidobacterium longum* subsp. *suillum* | 3 | -^a^ | 7 | 8 | 11 | 5 | 15 |
| 26 |  | *Bifidobacterium breve* | 28 | 5 | -^a^ | 45 | -^a^ | -^a^ | -^a^ |
| 27 |  | *Bifidobacterium pseudocatenulatum* | 22 | 9 | 11 | 47 | 2 | 4 | 14 |
| 28 |  | *Bifidobacterium animalis* subsp. *lactis* | 2 | 18 | 12 | 29 | 16 | -^a^ | 21 |
| 29 | *Staphylococcus* | *Staphylococcus epidermidis* | 8 | -^a^ | -^a^ | 9 | -^a^ | -^a^ | -^a^ |
| 30 |  | *Staphylococcus hyicus* | 1 | 2 | 2 | -^a^ | -^a^ | -^a^ | -^a^ |
| 31 |  | *Staphylococcus aureus* | 2 | 12 | 1 | 1 | - | -^a^ | 12 |
| 32 |  | *Staphylococcus simulans* | -^a^ | -^a^ | -^a^ | -^a^ | -^a^ | 8 | -^a^ |
| 33 | *Micrococcus* | *Micrococcus luteus* | -^a^ | -^a^ | -^a^ | -^a^ | -^a^ | 3 | -^a^ |
| 34 | *Actinomyces* | *Actinomyces radicidentis* | -^a^ | 9 | -^a^ | -^a^ | -^a^ | -^a^ | -^a^ |
| 35 | *Escherichia* | *Escherichia coli* | 5 | 12 | 18 | 5 | 28 | 2 | 8 |
| 36 | *Morganella* | *Morganella morganii* | -^a^ | -^a^ | -^a^ | -^a^ | -^a^ | 2 | 5 |
| 37 | *Weissella* | *Weissella confusa* | -^a^ | -^a^ | 5 | -^a^ | -^a^ | -^a^ | -^a^ |
| 38 | *Pediococcus* | *Pediococcus pentosaceus* | -^a^ | -^a^ | -^a^ | 12 | 34 | 22 | -^a^ |

^a^The strain was not isolated. Fb1: Fbkuang; Fb2: Fblai; Fb3: Fblei; Fb4: Fblin; Fb5: Fbliu; Fb6: Fbwang; Fb7: Fbzhao.

**Table S3 The optical density of all isolated strains of *B. pseudocatenulatum* after 48 h of growth (OD_600_ nm)**

| **No.** | **Centenarians** | **Strains** | **OD_600_ nm**^b^ |
| --- | --- | --- | --- |
| 1 | Fb1 | kuang-4 | 2.107 |
| 2 | Fb1 | kuang-5 | 1.984 |
| 3 | Fb1 | kuang-12 | 1.772 |
| 4 | Fb1 | kuang-15 | 1.929 |
| 5 | Fb1 | kuang-16 | 2.067 |
| 6 | Fb1 | kuang-45 | 2.165 |
| 7 | Fb1 | kuang-46 | **2.201** |
| 8 | Fb1 | kuang-47 | 2.113 |
| 9 | Fb1 | kuang-62 | 1.654 |
| 10 | Fb1 | kuang-71 | 1.874 |
| 11 | Fb1 | kuang-89 | 1.902 |
| 12 | Fb1 | kuang-152 | 1.987 |
| 13 | Fb1 | kuang-154 | 2.113 |
| 14 | Fb1 | kuang-157 | 1.494 |
| 15 | Fb1 | kuang-159 | 2.039 |
| 16 | Fb1 | kuang-160 | 2.034 |
| 17 | Fb1 | kuang-161 | 2.075 |
| 18 | Fb1 | kuang-182 | 2.175 |
| 19 | Fb1 | kuang-183 | 2.048 |
| 20 | Fb1 | kuang-189 | 1.979 |
| 21 | Fb1 | kuang-190 | 1.977 |
| 22 | Fb1 | kuang-191 | 2.133 |
| 23 | Fb2 | lai-12 | 2.156 |
| 24 | Fb2 | lai-15 | 2.100 |
| 25 | Fb2 | lai-18 | 1.783 |
| 26 | Fb2 | lai-28 | 1.929 |
| 27 | Fb2 | lai-49 | 2.081 |
| 28 | Fb2 | lai-50 | 2.174 |
| 29 | Fb2 | lai-82 | 2.090 |
| 30 | Fb2 | lai-84 | 2.086 |
| 31 | Fb2 | lai-85 | 2.073 |
| 32 | Fb3 | lei-1 | 2.126 |
| 33 | Fb3 | lei-12 | 2.166 |
| 34 | Fb3 | lei-13 | 1.858 |
| 35 | Fb3 | lei-32 | 1.477 |
| 36 | Fb3 | lei-36 | 2.182 |
| 37 | Fb3 | lei-75 | 2.084 |
| 38 | Fb3 | lei-77 | 1.998 |
| 39 | Fb3 | lei-89 | 2.102 |
| 40 | Fb3 | lei-162 | 2.139 |
| 41 | Fb3 | lei-163 | 2.045 |
| 42 | Fb3 | lei-165 | 1.783 |
| 43 | Fb4 | lin-2 | 2.112 |
| 44 | Fb4 | lin-3 | 2.155 |
| 45 | Fb4 | lin-5 | 2.156 |
| 46 | Fb4 | lin-12 | 2.073 |
| 47 | Fb4 | lin-18 (NCU-08)^a^ | **2.236** |
| 48 | Fb4 | lin-19 | 1.962 |
| 49 | Fb4 | lin-36 | 1.858 |
| 50 | Fb4 | lin-53 | 1.767 |
| 51 | Fb4 | lin-54 | 2.126 |
| 52 | Fb4 | lin-78 | 1.784 |
| 53 | Fb4 | lin-80 | 1.655 |
| 54 | Fb4 | lin-81 | 2.145 |
| 55 | Fb4 | lin-82 | 2.051 |
| 56 | Fb4 | lin-145 | 2.117 |
| 57 | Fb4 | lin-147 | 2.128 |
| 58 | Fb4 | lin-152 | 1.873 |
| 59 | Fb4 | lin-153 | **2.209** |
| 60 | Fb4 | lin-175 | 2.139 |
| 61 | Fb4 | lin-176 | 1.533 |
| 62 | Fb4 | lin-177 | 1.786 |
| 63 | Fb4 | lin-179 | 2.056 |
| 64 | Fb4 | lin-182 | 2.017 |
| 65 | Fb4 | lin-215 | 1.998 |
| 66 | Fb4 | lin-216 | 2.157 |
| 67 | Fb4 | lin-219 | 2.018 |
| 68 | Fb4 | lin-226 | 2.119 |
| 69 | Fb4 | lin-257 | **2.227** |
| 70 | Fb4 | lin-258 | 1.882 |
| 71 | Fb4 | lin-262 | 1.974 |
| 72 | Fb4 | lin-264 | 1.999 |
| 73 | Fb4 | lin-267 | 2.084 |
| 74 | Fb4 | lin-269 | 2.145 |
| 75 | Fb4 | lin-281 | 2.176 |
| 76 | Fb4 | lin-282 | 2.080 |
| 77 | Fb4 | lin-285 | 2.068 |
| 78 | Fb4 | lin-286 | 1.974 |
| 79 | Fb4 | lin-287 | 2.165 |
| 80 | Fb4 | lin-297 | 2.118 |
| 81 | Fb4 | lin-298 | 2.185 |
| 82 | Fb4 | lin-300 | 2.038 |
| 83 | Fb4 | lin-309 | 2.115 |
| 84 | Fb4 | lin-310 | 2.076 |
| 85 | Fb4 | lin-311 | 1.937 |
| 86 | Fb4 | lin-314 | 2.065 |
| 87 | Fb4 | lin-316 | 2.139 |
| 88 | Fb4 | lin-318 | 2.115 |
| 89 | Fb4 | lin-319 | 2.045 |
| 90 | Fb5 | liu-73 | 1.652 |
| 91 | Fb5 | liu-76 | 2.136 |
| 92 | Fb6 | wang-2 | 1.892 |
| 93 | Fb6 | wang-5 | 1.671 |
| 94 | Fb6 | wang-37 | 2.173 |
| 95 | Fb6 | wang-52 | 2.082 |
| 96 | Fb7 | zhao-8 | 2.107 |
| 97 | Fb7 | zhao-10 | **2.194** |
| 98 | Fb7 | zhao-13 | 1.879 |
| 99 | Fb7 | zhao-18 | 2.075 |
| 100 | Fb7 | zhao-19 | 1.574 |
| 101 | Fb7 | zhao-39 | 1.563 |
| 102 | Fb7 | zhao-47 | 1.298 |
| 103 | Fb7 | zhao-52 | 2.016 |
| 104 | Fb7 | zhao-53 | 1.753 |
| 105 | Fb7 | zhao-55 | 1.986 |
| 106 | Fb7 | zhao-57 | 2.108 |
| 107 | Fb7 | zhao-82 | 2.148 |
| 108 | Fb7 | zhao-89 | 2.123 |
| 109 | Fb7 | zhao-95 | 1.702 |

Note: ^a^lin-18: The name of the original isolated strains; NCU-08: The name of the patent deposited by the lin-86 strain (China Microbial Culture Collection Center) (CGMCC No.26490). ^b^The data for absorbance are presented as mean (n=3). Fb1: Fbkuang; Fb2: Fblai; Fb3: Fblei; Fb4: Fblin; Fb5: Fbliu; Fb6: Fbwang; Fb7: Fbzhao.

**Table S4 16S rRNA sequence of** ***B. pseudocatenulatum* NCU-08**

| **Strain** | **Sequence (350 bp)** |
| --- | --- |
| NCU-08 | GGGTGAGTAATGCGTGACCGACCTGCCCCATACACCGGAATAGCTCCTGGAAACGGGTGGTAATGCCGGATGCTCCGACTCCTCGCATGGGGTGTCGGGAAAGATTTCATCGGTATGGGATGGGGTCGCGTCCTATCAGGTAGTCGGCGGGGTAACGGCCCACCGAGCCTACGACGGGTAGCCGGCCTGAGAGGGCGACCGGCCACATTGGGACTGAGATACGGCCCAGACTCCTACGGGAGGCAGCAGTGGGGAATATTGCACAATGGGCGCAAGCCTGATGCAGCGACGCCGCGTGCGGGATGACGGCCTTCGGGTTGTAAACCGCTTTTGATCGGGAGCAAGCCTTC |

Note: This sequence is a cropped sequence used for alignment in NCBI.


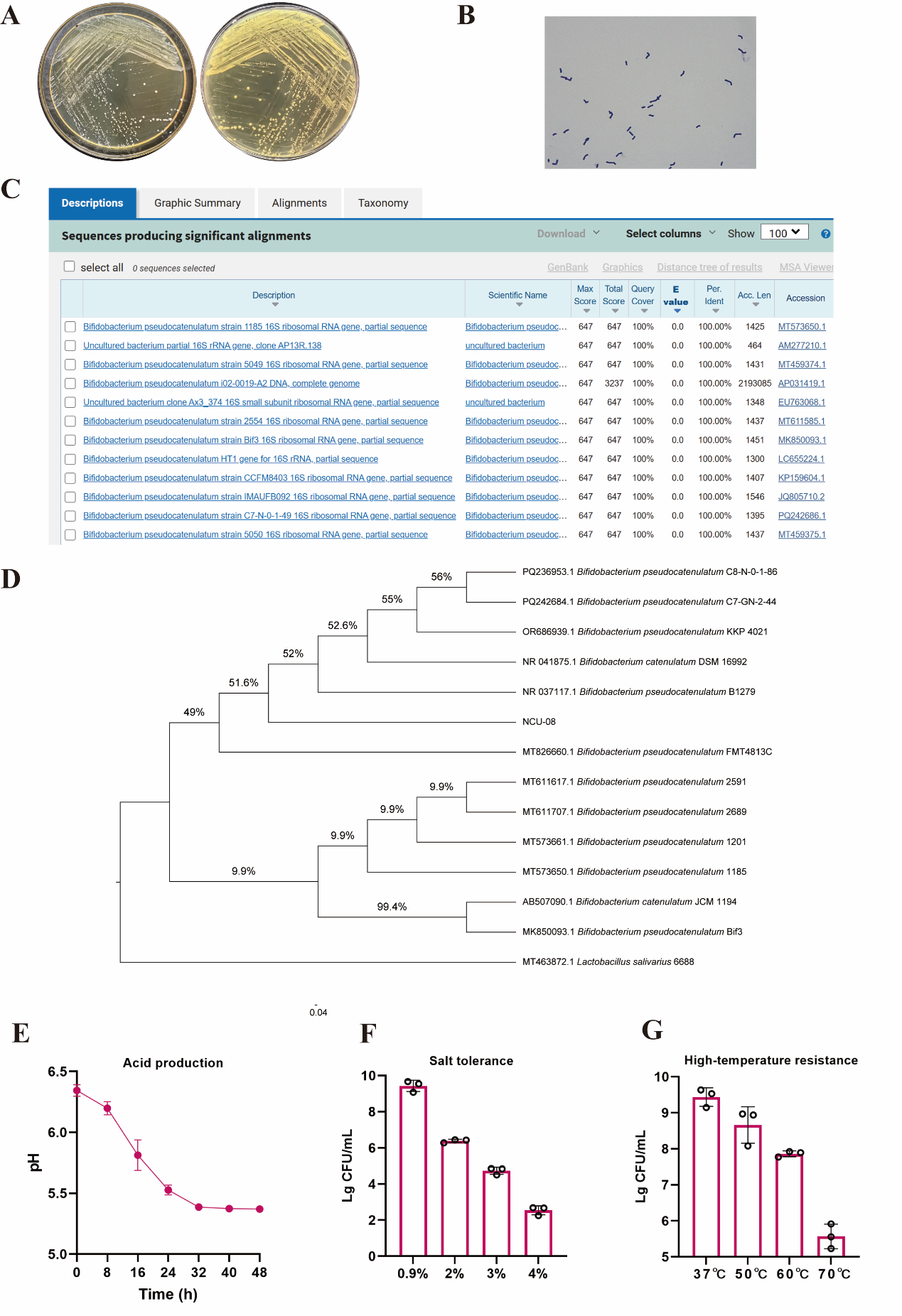


**Fig. S2 Identification and evaluation of probiotic functions from *B. pseudocatenulatum* NCU-08.** (A) Plate colony morphology (front vs. back). (B) Crystal violet staining morphology (oil mirror, 100x). (C) Sequence alignment in NCBI data. (D) Phylogenetic tree constructed by the neighbor-joining method. (E) Acid production (n=3). (F) Salt tolerance (NaCl, n=3). (G) high-temperature resistance (n=3). Data are presented as mean ± SD.


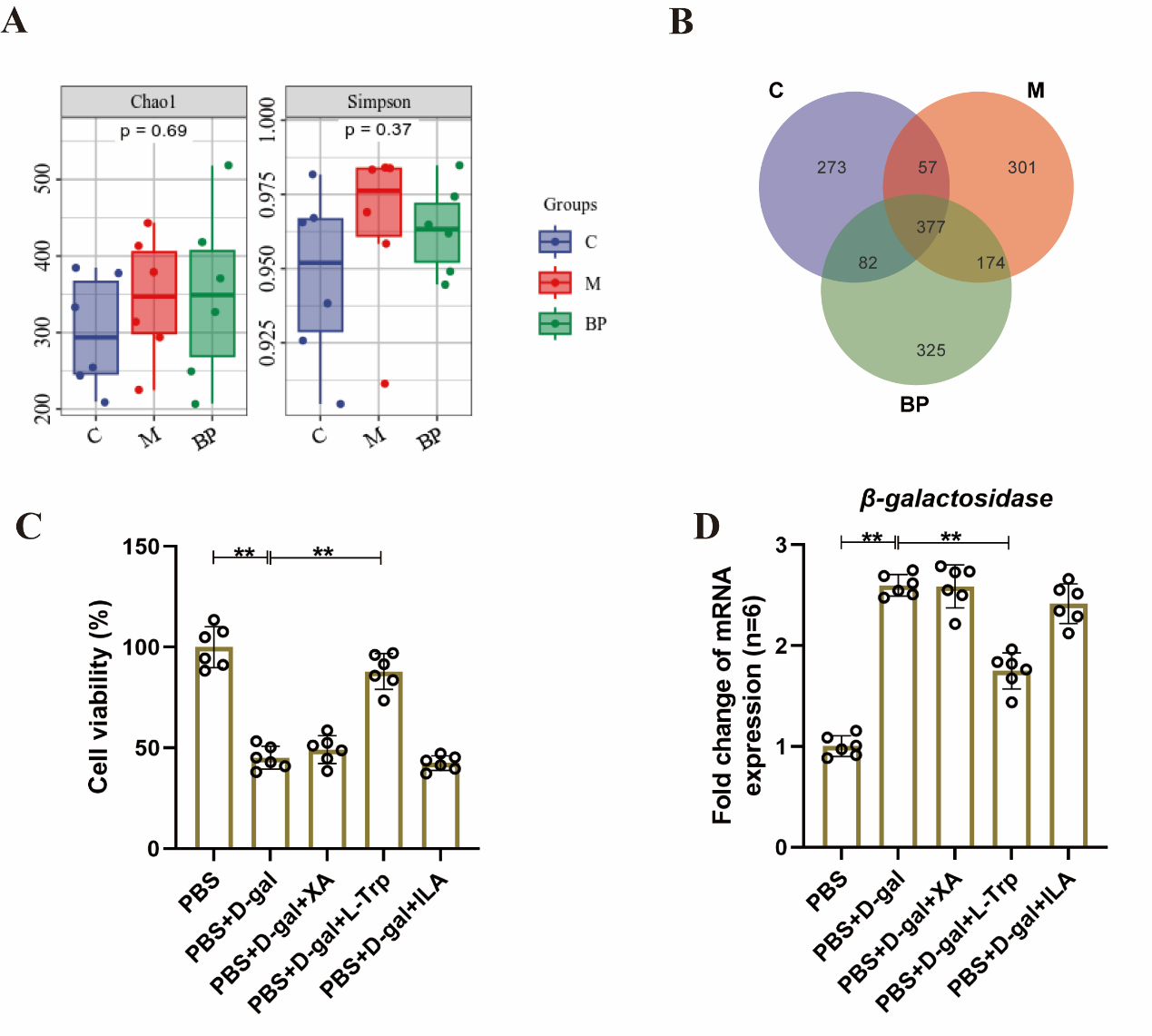


**Fig. S3 *B. pseudocatenulatum* NCU-08 improved the composition of gut microbiota and three metabolites for anti-aging evaluation.** (A) Chao1 and Simpson indices. (B) Venn diagram. (C) Cell viability test (n=6). (D) RT-qPCR was used to detect the transcription level of *β-galactosidase* gene (n=6). D-gal: D-galactose. XA: Xanthurenic acid. L-Trp: L-tryptophan. ILA: Indole-3-lactic acid. C: Control group. M: Model group. BP: *B. pseudocatenulatum* NCU-08 group. Data are presented as mean ± SD, and analyzed using one-way ANOVA for statistical significance, followed by Tukey’s test for multiple comparisons. Significance levels are indicated as ***p* < 0.01.

**Table S5 Levels of tryptophan metabolites in feces of mice from each group (ng/mL)**

| **Compounds** | **C1** | **C2** | **C3** | **C4** | **M1** | **M2** | **M3** | **M4** | **BP1** | **BP2** | **BP3** | **BP4** |
| --- | --- | --- | --- | --- | --- | --- | --- | --- | --- | --- | --- | --- |
| 2-Aminophenol | 895.530801 | 642.033774 | 582.797303 | 596.771745 | 656.904809 | 587.385217 | 660.563636 | 616.559278 | 795.050547 | 877.131895 | 763.424194 | 481.367538 |
| 2-Aminobenzoic acid | 26.4687885 | 102.393329 | 82.6721496 | 80.9877408 | 17.6509005 | 24.3592753 | 44.9278469 | 60.7290841 | 69.7257066 | 63.9377852 | 129.134501 | 64.6594507 |
| 2-Amino-3-methoxybenzoic acid | N/A | N/A | N/A | N/A | N/A | N/A | N/A | N/A | N/A | N/A | N/A | N/A |
| 3-Hydroxyanthranilic acid | N/A | N/A | N/A | N/A | N/A | N/A | N/A | N/A | N/A | N/A | N/A | N/A |
| 3-Indoleglyoxylic acid | N/A | N/A | N/A | N/A | N/A | N/A | N/A | N/A | N/A | N/A | N/A | N/A |
| 5-Methoxyindoleacetic acid | N/A | N/A | N/A | N/A | N/A | N/A | N/A | N/A | N/A | N/A | N/A | N/A |
| 5-Hydroxyindoleacetic acid | N/A | N/A | N/A | N/A | N/A | N/A | N/A | N/A | N/A | N/A | N/A | N/A |
| 6-Hydroxy melatonin | 384.73306 | N/A | N/A | N/A | N/A | 522.563311 | 347.090909 | N/A | 592.348876 | N/A | N/A | N/A |
| Tryptophol | 44.3679671 | 159.845815 | N/A | N/A | 77.5377993 | N/A | 80.402201 | N/A | N/A | N/A | N/A | N/A |
| Indole-3-acetamide | N/A | 38.725194 | 21.2412137 | 22.2674645 | N/A | N/A | N/A | 18.0972443 | N/A | 23.2938615 | 33.6047553 | 21.1939729 |
| 5-Hydroxytryptophol | 25.3721766 | N/A | 12.5238047 | 14.0457287 | 10.4110429 | 12.0694873 | 8.52600957 | 8.50395519 | 10.3265938 | 8.3990253 | 7.08971349 | 8.19985695 |
| Tryptamine | 6.33510267 | 49.4767149 | 2.0709338 | 2.62609457 | 35.423016 | 10.387379 | 64.4026794 | 9.15640365 | 5.46283268 | 7.76435089 | 5.43465977 | 14.6044249 |
| Melatonin | N/A | N/A | N/A | N/A | N/A | N/A | N/A | N/A | N/A | N/A | N/A | N/A |
| Hydroxytryptophan | 62.9727926 | 118.933291 | 101.652329 | 112.535513 | 51.3667128 | 69.7137122 | 70.6423923 | 108.635012 | 93.8791005 | 113.733928 | 118.863908 | 89.8960519 |
| N-acetylserotonin | N/A | N/A | N/A | N/A | N/A | N/A | N/A | N/A | N/A | N/A | N/A | N/A |
| Serotonin | 846.459959 | 565.656597 | 722.599101 | 892.412921 | 312.532159 | 432.064031 | 326.096651 | 361.32732 | 528.590881 | 545.66881 | 569.302626 | 431.37326 |
| L-tryptophan | 18261.0883 | 23059.786 | 15383.1222 | 19291.4964 | 5989.8872 | 2913.45481 | 6436.01914 | 6520.59006 | 14313.0452 | 14588.7599 | 12904.7951 | 17707.89624 |
| Indolylpropionic acid | 1345.52361 | 266.491504 | 288.536984 | 352.272816 | 311.6792 | 225.14824 | 323.973206 | 258.88184 | 179.695688 | 908.934052 | 590.102467 | 333.898531 |
| Indole-3-lactic acid | 2372.24846 | 1319.32033 | 498.878218 | 443.278848 | 93.500099 | 202.17727 | 158.663158 | 229.516257 | 144.582216 | 118.88428 | 428.628134 | 774.2428 |
| Indole-3-carbaldehyde | 119.400411 | 74.4873086 | 56.8874132 | 61.6693909 | 45.7808233 | 34.7256537 | 54.1372249 | 59.024088 | 30.1262637 | 161.00477 | 104.389176 | 44.5263208 |
| β-Indole-3-acetic acid | 1625.02053 | 2250.3776 | 3444.74867 | 4200.88539 | 3227.44904 | 3806.37225 | 3012.37321 | 3061.46907 | 1579.27584 | 3850.23849 | 2689.52447 | 3254.64429 |
| Indole-3-β-acrylic acid | 37.1405544 | 22.9787078 | 16.3225378 | 24.6498346 | 14.2634079 | 14.8847025 | 18.9538756 | 13.8357454 | 9.549804 | 33.8598092 | 24.3730601 | 13.431337 |
| 2-Ketoadipic acid | 70.2856263 | 48.9320327 | 51.3745403 | N/A | N/A | N/A | N/A | 80.7152062 | 53.7845059 | 57.5095396 | 87.9560287 | N/A |
| N-formylkynurenine | 85.973614 | 242.30858 | 151.15141 | 129.638062 | 54.9703147 | 85.4519251 | 70.9887081 | 92.9399286 | 57.8572313 | 124.139361 | 213.769399 | 82.1457181 |
| L-kynurenine | 9.76340862 | 16.4544787 | 11.8857785 | 12.1026464 | 8.63685929 | 9.52845378 | 7.97960766 | 9.81639572 | 10.7591294 | 10.0168187 | 10.3964385 | 12.7368873 |
| Cinnabarinic acid | 15.900616 | 8.42337948 | 8.32471394 | 13.1064409 | 9.77151197 | 13.315421 | 8.84403828 | 10.0777161 | 14.9709098 | 12.9636043 | 13.5448667 | 9.11016594 |
| Picolinic acid | 41715.4004 | 23195.4059 | 17878.1161 | 20212.0062 | 25638.7295 | 17179.6376 | 29643.6364 | 14748.5131 | 16620.2806 | 49228.9506 | 26467.9666 | 13822.5253 |
| Nicotinic acid | 16633.4702 | 11422.6977 | 10170.9338 | 9741.87585 | 12910.0534 | 10155.3016 | 12273.5885 | 8825.47581 | 12226.5319 | 16250.7258 | 13880.4218 | 8602.35552 |
| Quinolinic acid | 1108.05955 | 1453.5557 | 1804.48508 | 2480.31718 | 1568.78092 | 2250.77208 | 1724.14354 | 1432.75178 | 2288.43615 | 2871.96184 | 2283.69479 | 1846.84341 |
| Kynurenic acid | N/A | 694.481854 | 1115.83572 | 1239.53104 | N/A | N/A | N/A | 831.919112 | N/A | N/A | 1261.69916 | 832.407019 |
| Xanthurenic acid | 1732.22793 | 3962.35578 | 7342.6747 | 6751.29403 | 609.00752 | 677.45419 | 1572.52632 | 3922.24425 | 3982.11265 | 3383.55454 | 8392.79745 | 2693.01926 |

N/A: The sample is not detected or the data is not available.


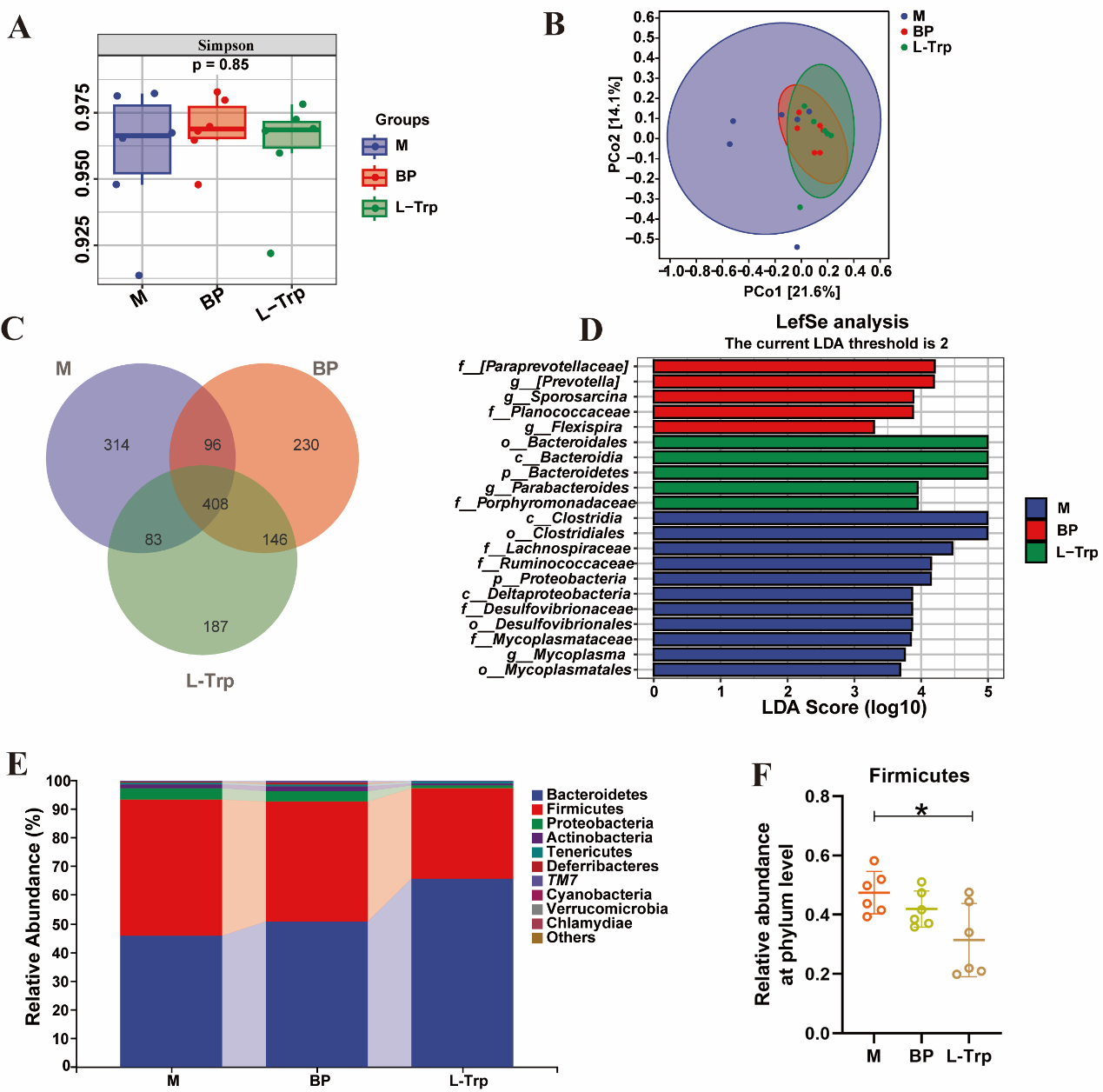


**Fig. S4 The L-tryptophan improved some characteristics of the gut microbiota in SAMP8 mice.**

(A) Simpson index. (B) PCoA analysis. (C) Venn diagram. (D) Linear Discriminant Analysis (LEFSe) analysis. (E) Taxonomic composition at the phylum level. (F) Relative abundance of Firmicutes. M: Model group. BP: *B. pseudocatenulatum* NCU-08 group. L-Trp: L-tryptophan group. Data are presented as mean ± SD, and analyzed using one-way repeated measures ANOVA, followed by Tukey’s test for multiple comparisons. Significance levels are indicated as **p* < 0.05.


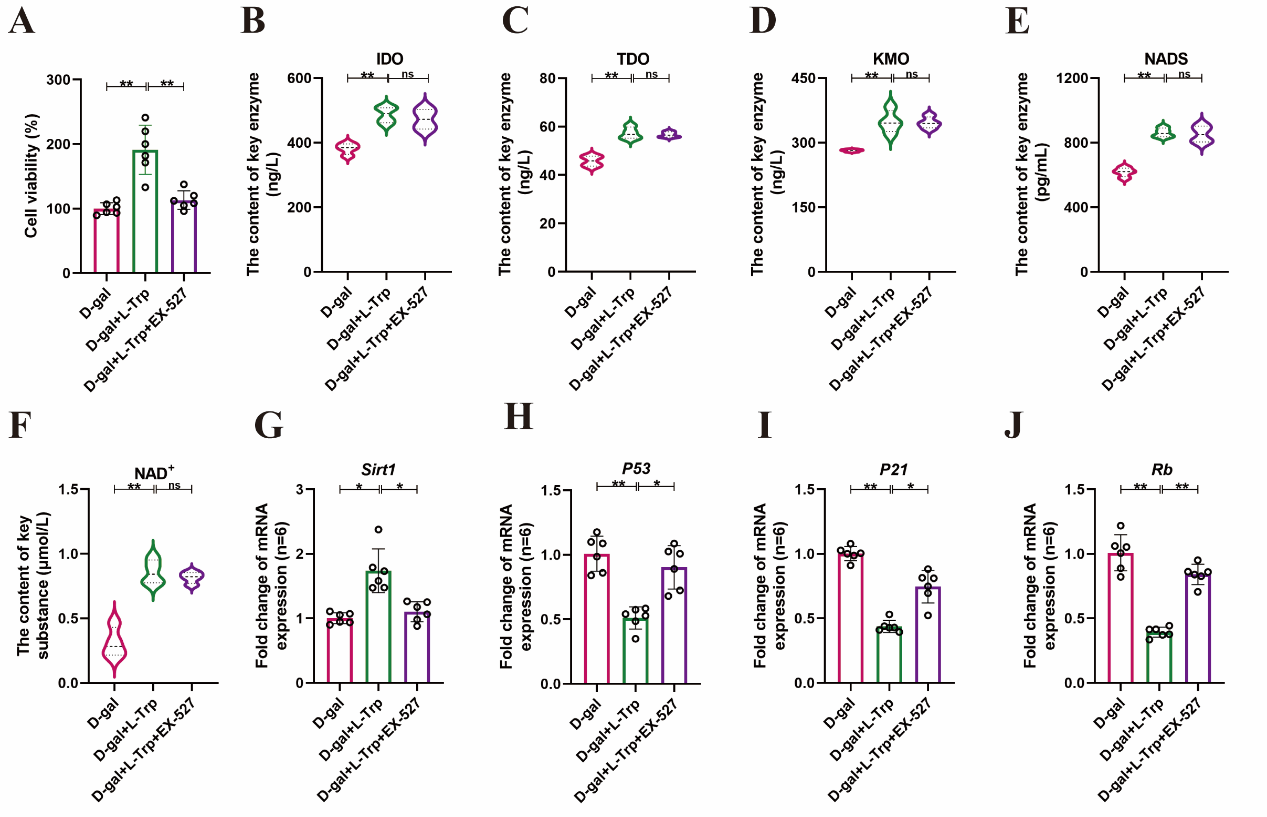


**Fig. S5 EX-527 confirmed that T-Lrp effectively improved cell activity, levels of related enzymes and transcription factors.** (A) Cell viability test. (B-E) Levels of key enzymes IDO, TDO, KMO, and NADS in the tryptophan metabolism pathway (n=4). (F) Determination of NAD^+^ content (n=4). (G-J) mRNA level of *Sirt1*, *P53*, *P21* and *Rb* (n=6). IDO: Indoleamine 2,3-dioxygenase. TDO: Tryptophan 2,3-dioxygenase. KMO: Kynurenine 3-monooxygenase. NADS: NAD synthetase. NAD^+^: Nicotinamide adenine dinucleotide. D-gal: D-gal group. D-gal+ L-Trp: D-gal+ L-tryptophan group. D-gal+ L-Trp+EX-527: D-gal+ L-tryptophan+EX-527 group. Data are presented as mean ± SD, and analyzed using one-way repeated measures ANOVA, followed by Tukey’s test for multiple comparisons. Significance levels are indicated as **p* < 0.05 and ***p* < 0.01. “ns” was no significant.


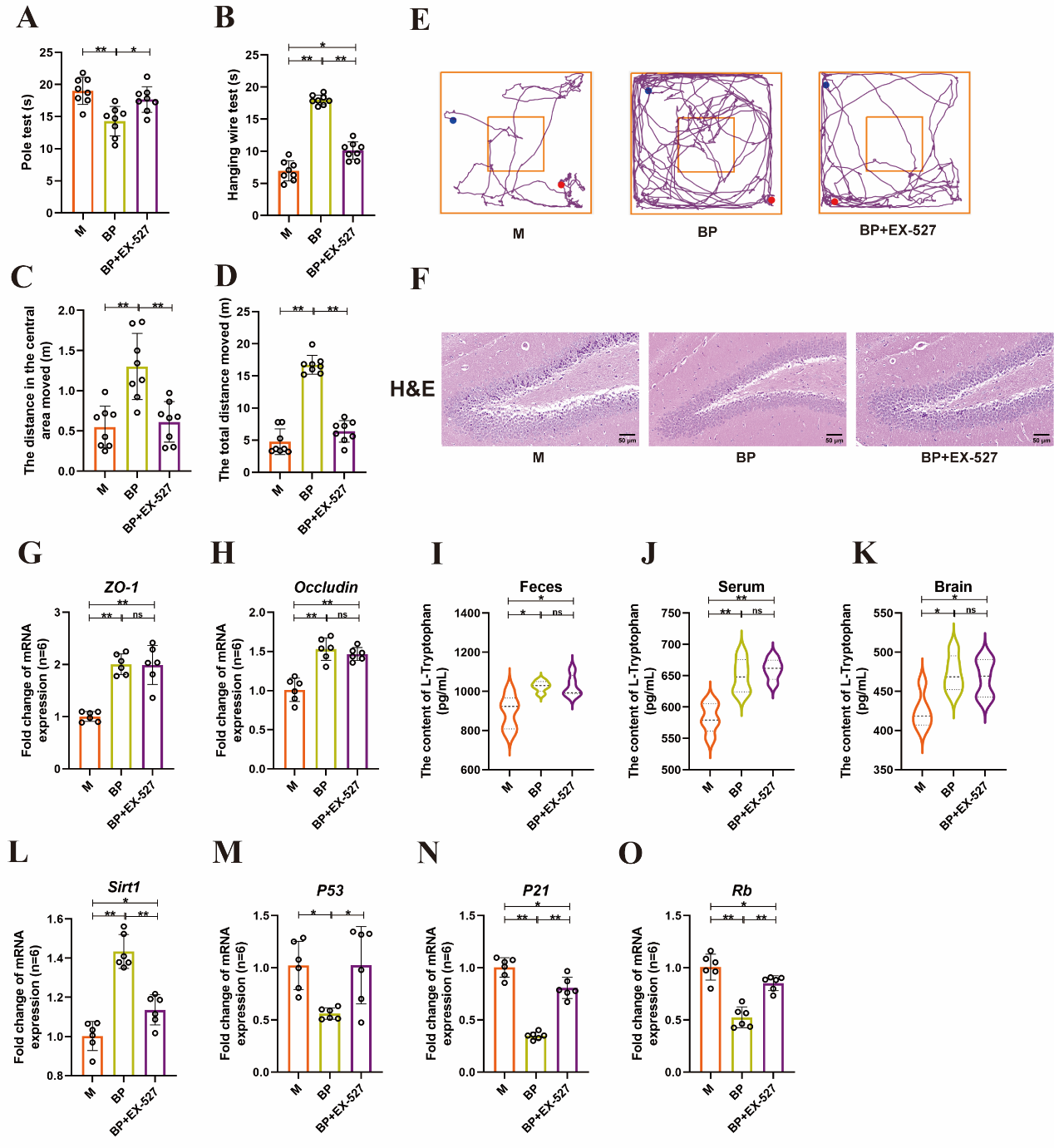


**Fig. S6 EX-527 confirmed that *B. pseudocatenulatum* NCU-08 had an effect on ameliorating senescence.** (A) Pole test (s). (B) Hanging wire test (s). (C) The distance to move into the central area in five minutes (m). (D) The total distance to move in five minutes (m). (E) The trajectories of movement by mice. (F) H&E staining of the hippocampus region (50 μm). (G) The mRNA level of *ZO-1* in intestinal tissue (n=6). (H) The mRNA level of *Occludin* in intestinal tissue (n=6). (I) The content of L-tryptophan in feces (n=5). (J) The content of L-tryptophan in serum (n=5). (K) The content of L-tryptophan in brain tissue (n=5). (L-O) The mRNA level of *Sirt1*, *P53*, *P21* and *Rb* (n=6). M: Model group. BP: *B. pseudocatenulatum* NCU-08 group. BP+EX-527: *B. pseudocatenulatum* NCU-08+EX-527 group. Data are presented as mean ± SD, and analyzed using one-way repeated measures ANOVA, followed by Tukey’s test for multiple comparisons. Significance levels are indicated as **p* < 0.05 and ***p* < 0.01. “ns” was no significant.


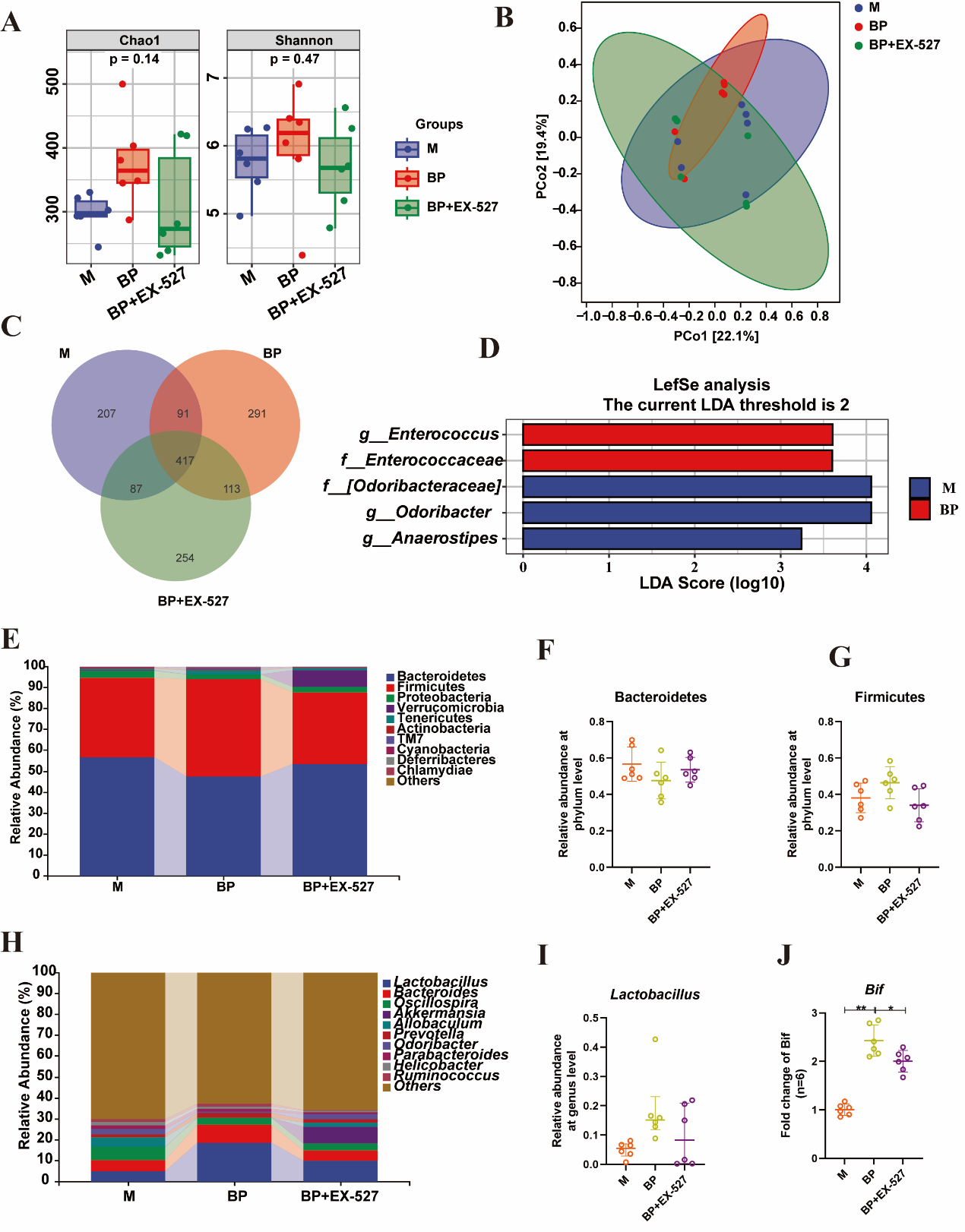


**Fig. S7 EX-527 confirmed that *B. pseudocatenulatum* NCU-08 improved the composition of gut microbiota in SAMP8 mice.** (A) Chao1 and Shannon indices. (B) PCoA analysis. (C) Venn diagram. (D) LEFSe analysis. (E) Taxonomic composition at the phylum level. (F-G) Relative abundance of Bacteroidetes and Firmicutes. (H) Microbial taxonomic composition at the genus level. (I) Relative abundance of *Lactobacillus*. (J) RT-qPCR detection of *Bif* (*Bifidobacterium*) expression in feces. M: Model group. BP: *B. pseudocatenulatum* NCU-08 group. BP+EX-527: *B. pseudocatenulatum* NCU-08+EX-527 group. Data are presented as mean ± SD or median (interquartile range), and analyzed using one-way repeated measures ANOVA, followed by Tukey’s test for multiple comparisons. Significance levels are indicated as **p* < 0.05 and ***p* < 0.01.
